# Supplementary material for: An approximate stochastic optimal control framework to simulate nonlinear neuro-musculoskeletal models in the presence of noise
Source: PLoS Comput Biol. 2022 Jun 8;18(6):e1009338. doi: 10.1371/journal.pcbi.1009338 (PMC9176817; doi:10.1371/journal.pcbi.1009338)
Supplement: S1 Text — (PDF) [file pcbi.1009338.s001.pdf]

Supplementary Information

1. Supplementary results and discussion

Mean unperturbed reach trajectories and muscle activations

Here, we give a more detailed description of the simulated mean reach trajectories and mean muscle activations. The simulated mean trajectories for the circle and bar are slightly curved (Figure 1A) as has been observed experimentally [1]. In the obstacle task, there is very little curvature in the first part of the reaching trajectory, whereas the motion of the hand was constrained to be on a straight vertical line in the simulation to mimic the presence of an obstacle. 95% confidence ellipses show the estimated position distribution for the optimal movement trajectory every 10ms (Figure 1A). The simulated hand position variability is in line with the minimum intervention principle, i.e. variability in the horizontal direction is controlled in the circle but not in the bar task. Since the reaching trajectories are alike for the different tasks, the mean tangential hand velocities are similar as well (Figure 1B). They exhibit the experimentally observed bell shapes [1], [2] and realistic maximal velocities for this reaching task [3]. Finally, the mean muscle activations are similar as well for the three reaching tasks. In the obstacle task the uniarticular elbow muscles are coordinated slightly different to match the kinematic task constraint for the mean hand trajectory. This potentially explains the slight increase in co-contraction for this task as compared to the circle and bar task.

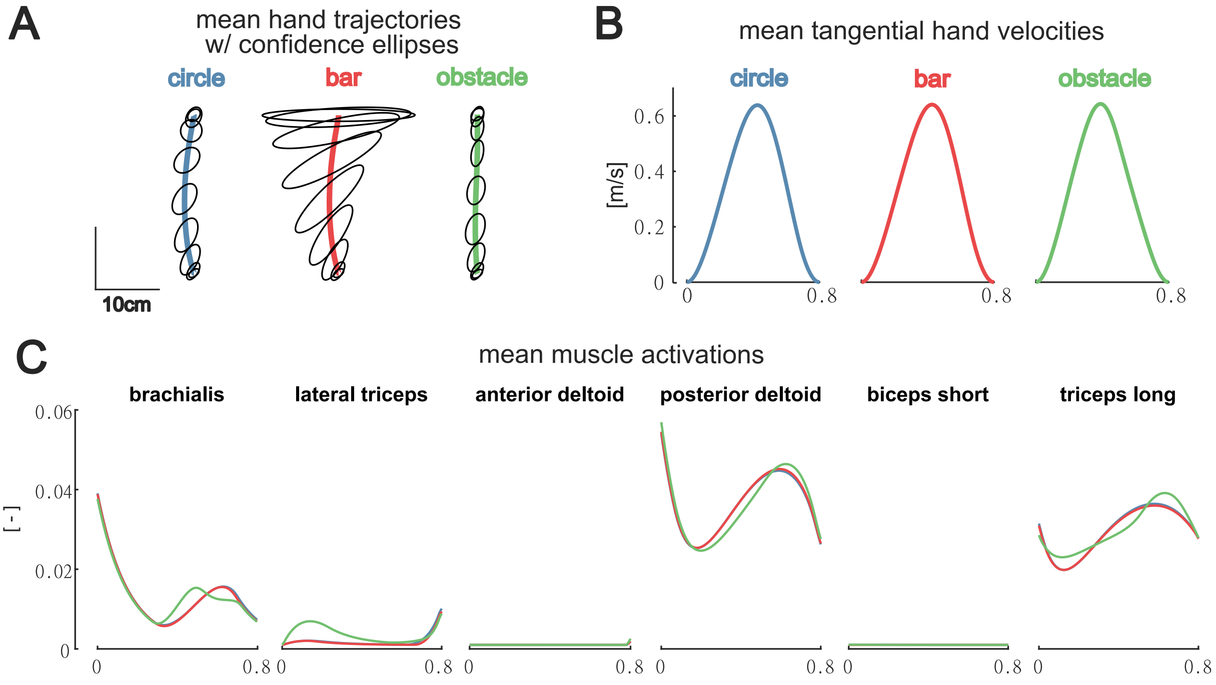

Figure 1 - A) Optimal mean hand trajectories for the three reaching tasks with 95% confidence ellipses every 10ms during the movement. B) Optimal tangential hand velocity profiles of the mean reaching movement. C) Mean muscle activations of the six different muscles for the different reaching targets. The 'circle' (blue) and 'bar' (red) activations often overlap.

Effect of biarticular muscles and muscle properties

While developing the simulations, we noticed that the arm model and especially muscle properties could largely affect the reach trajectories. We therefore decided to explore how the presence of biarticular muscles and muscle properties affected simulated unperturbed and perturbed reach

trajectories and muscle activations. We performed simulations with three models (Figure 2 and Figure 3). Model 1 (Todorov2003 – 6 muscles) is the model presented in the main text. Model 2 (Todorov2003 – 4 muscles) was derived from model 1 by removing the biarticular muscles. Model 3 (adapted model – 4 muscles) also contained only mono-articular muscles and was derived from a more complex musculoskeletal model ([4], [5]). Model 3 differs from models 1 and 2 in its moment arms, muscle-tendon lengths and maximal isometric muscle forces (Table 1). In addition, the force-length relationship in model 3 was shifted such that normalized fiber lengths remained within a plausible operating range, i.e. remained below normalized fiber lengths at which huge passive forces would be developed, for the full range of motion for the movement. To this aim, we evaluated the force-length relationship for $\tilde{l}_{M,shifted}$, where $\tilde{l}_{M,shifted} = 0.5\tilde{l}_M + 0.3$.

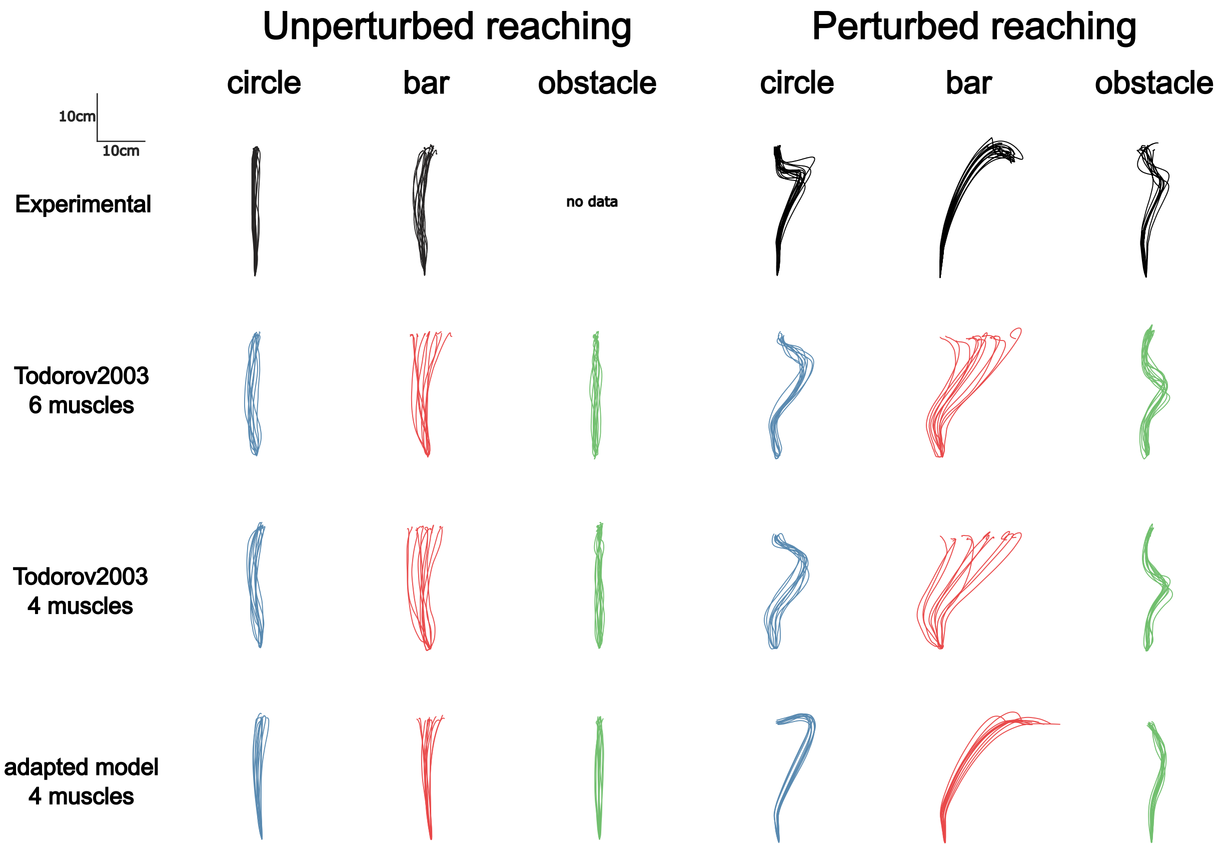

Figure 2 - Unperturbed and perturbed reach trajectories resulting from stochastic optimal controllers of three different models.

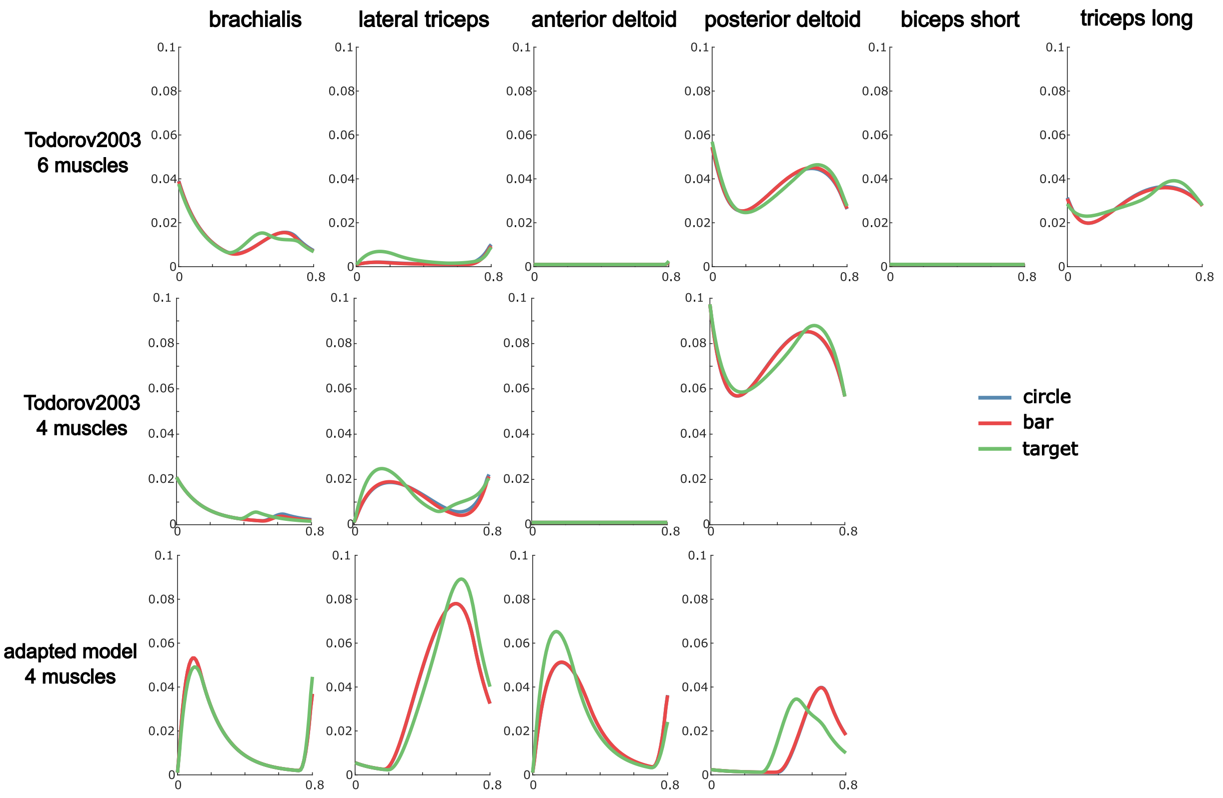

Figure 3 - Mean muscle activations during reaching to different targets for the different models

Removing the biarticular muscles (model 1 → model 2) altered the mean muscle activations but had little effect on the reaching trajectories (Figure 2). During unperturbed reaches, the kinematic behavior of the models with and without biarticular muscles is strikingly similar (Figure 2, unperturbed reaching). Even in the presence of perturbations, reaching trajectories are very similar for both models for all three reaching tasks (Figure 2, perturbed reaching).

Altering the muscle-tendon properties (model 2 → model 3) had a large effect on both mean muscle activations and reaching trajectories (Figure 2 and Figure 3). Reaching trajectories were less variable for model 3 than for model 2, especially for the bar condition (Figure 2, unperturbed and perturbed reaching). Model 3 predicted later corrections to perturbations resulting in an overshoot of the target for the circle and bar conditions (Figure 2, perturbed reaching). This overshoot as observed in model 3 was in agreement with experimental data for the bar condition but not for the circle condition.

The implementations of all three models can be found in the supplemented code.

Table 1 - Difference in maximal muscle strength for three models.

$F_{ISO}[N]$						
	BRACH	LATTRI	ANTDEL	POSTDEL	BIC	LONGTRI
Model 1	572	445	700	382	159	318
Model 2	572	445	700	382	-	-
Model 3	1143	260	718	525	-	-

2. Rigid tendon assumption

In this section we aim to provide more insight in how the rigid tendon assumption might have affected the results. Note that using rigid tendons resulted in algebraic contraction dynamics and simplified the

optimal control problems. However, the framework is sufficiently general to consider compliant tendons in future work. Adding a compliant tendon in series to the muscle fibers will reduce the compliance of the muscle-tendon unit. This will have a large influence on muscle-tendon stiffness when the muscle is stiffer than the tendon as in our simulations of standing balance with short-range stiffness, but a small influence on muscle-tendon stiffness when the muscle is more compliant than the tendon as in our simulations of standing balance and reaching with a Hill-type muscle model. Here, we illustrate this for the soleus muscle. We chose the soleus, as it has a longer and thus more compliant tendon than the other muscles we modeled. Therefore, the effect of adding a compliant tendon is expected to be largest for this muscle.

We computed stiffness of the muscle fibers as $k_{muscle} = \frac{\partial F_m}{\partial l_m} \cos(\alpha)$ with F_m the muscle fiber force, l_m the muscle fiber length and α the pennation angle; stiffness of the tendon as $k_{tendon} = \frac{\partial F_T}{\partial l_T}$ with F_T the tendon force, l_T the tendon length. Stiffness of the muscle-tendon unit was computed as: $k_{MT} = \left(\frac{1}{k_{tendon}} + \frac{1}{k_{muscle}}\right)^{-1}$. Note that when assuming a rigid tendon $k_{MT} = k_{muscle}$.

We first discuss the observations for a soleus Hill-type muscle without short-range-stiffness. In the rigid tendon case, the length of both the fiber and tendon is the same for every activation level (Figure 4A – length, dotted lines). In the rigid tendon case, k_{muscle}, and thus k_{MT}, increases linearly with muscle activation level as the muscle is operating on the ascending limb of the force-length relationship (Figure 4A - stiffness, green dotted line). In the compliant tendon case, the fiber length decreases for increasing activation, changing the operating point of the muscle on the force-length relation, and the tendon length increases for increasing activation (Figure 4 A – length, solid lines). As a consequence, k_{muscle} (Figure 4A – stiffness, blue solid line) increases at a superlinear rate with activation level as muscle fiber length decreases with increasing activation level shifting the muscle's operating length to a steeper part of the force-length relation in this specific case. However, differences in muscle stiffness when considering a compliant instead of a rigid tendon are small Figure 4A – detail MT stiffness). The compliant tendon (Figure 4A – stiffness, red solid line) is much stiffer than the muscle fibers (at least factor 6), and as a result has a very minor effect on k_{MT} (green solid line). In Figure 4A – detail MT stiffness, we see that at high muscle activations (above 0.75), muscle-tendon stiffness becomes higher for the compliant than for the rigid tendon, because the increase in fiber stiffness due to fiber shortening outweighs the effect of the compliant tendon.

In the extended Hill-type model where we added activation dependent stiffness to the muscle fibers modeling short-range-stiffness, the addition of a compliant tendon has a large influence on muscle-tendon stiffness as the muscle fibers become much stiffer than the tendon due to the added element (compare Figure 4 A vs Figure 4B - stiffness). Note that even with the inclusion of a compliant tendon, modeling short-range stiffness results in about a five-fold increase in stiffness of the muscle-tendon unit at an activation level of 0.5 (Figure 4A vs Figure 4B, detail MT stiffness). We therefore expect simulations based on the muscle model with short-range-stiffness to change when including a compliant tendon. We speculate that for platform rotations in healthy individuals, postural sway might be lower as their strategy is to move the ankle in anti-phase to the platform which should be facilitated by a more compliant muscle-tendon unit. For vestibular loss subjects that try to follow the platform motion with their body a decrease in muscle-tendon stiffness might lead to lower levels of co-contraction, as it becomes less efficient, and higher postural sway. In the simulations of platform translations muscle co-contraction becomes a less efficient strategy at lower muscle tendon stiffness and would thus decrease for both healthy and vestibular loss subjects. This would then lead to an increase in postural sway for the translations.

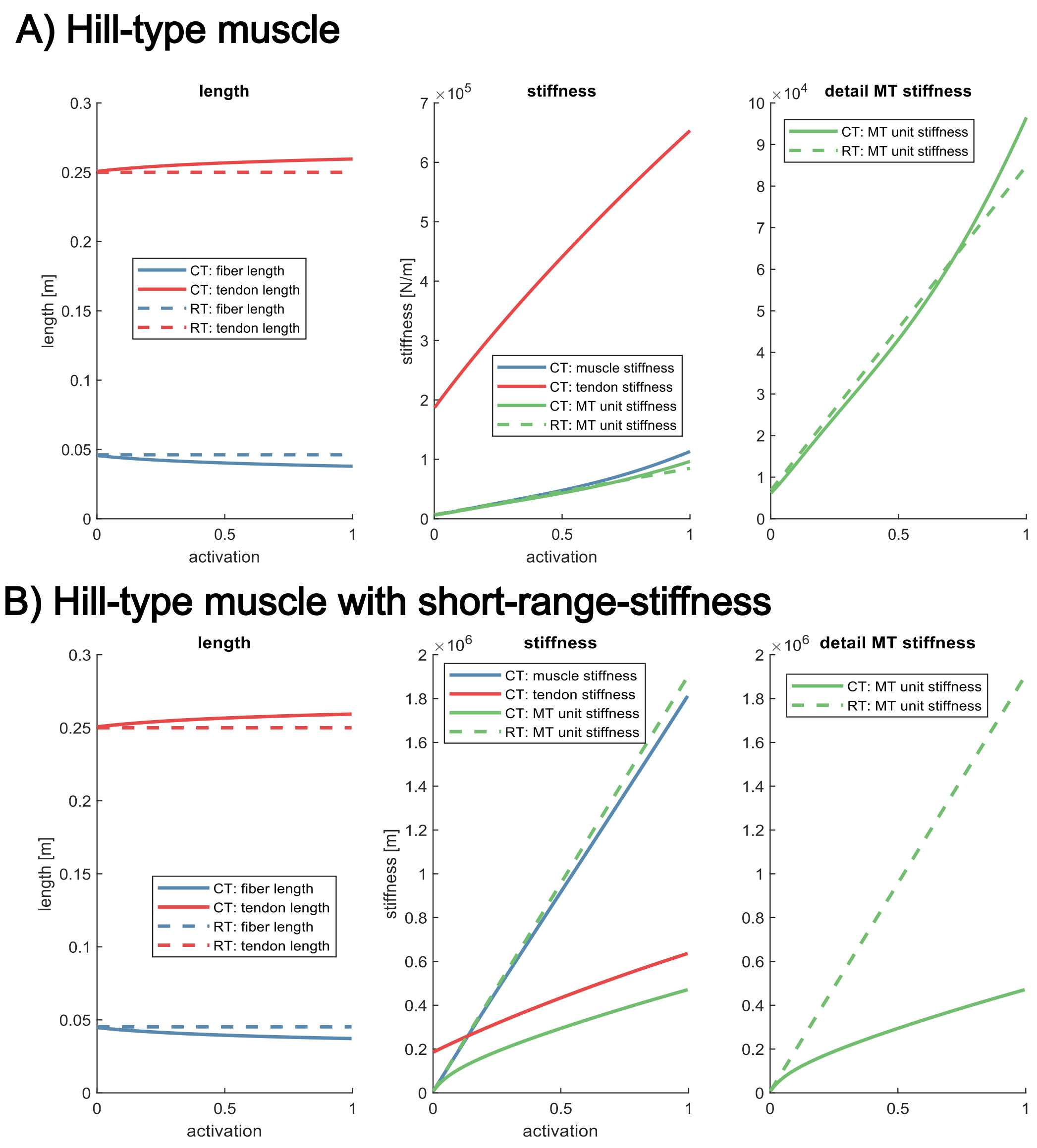

Figure 4 - simulated experiment for an isometric soleus muscle-tendon unit for different activation levels in the case of including a compliant tendon (CT) or a rigid tendon (RT) and in case of modelling the Hill-type muscle without (A) or with short-range-stiffness (B).

3. Supplementary methods

Approximate stochastic optimal control

We formulate predictive simulations of human movement as stochastic optimal control problems of the following form:

$$\min_{\boldsymbol{e}_{ff}(t),\boldsymbol{K}(t)} \quad J = E\left[\int \boldsymbol{e}^T(t)\boldsymbol{e}(t)dt\right]. \quad (1)$$

$$\text{subject to} \quad \dot{\boldsymbol{x}}(t) = \boldsymbol{f}\left(\boldsymbol{x}(t), \boldsymbol{e}(t), \boldsymbol{w}_{sys}\right) \quad \text{(stochastic dynamics)} \quad (2)$$

$$\boldsymbol{e}(t) = \boldsymbol{e}_{ff}(t) + \boldsymbol{K}(t) * \boldsymbol{y}_{fb}\left(\boldsymbol{x}(t), \boldsymbol{w}_{policy}\right) \quad \text{(control policy)} \quad (3)$$

$$(4)$$

$$\boldsymbol{g}\left(\boldsymbol{x}(t), \boldsymbol{e}_{ff}(t), \boldsymbol{K}(t), \boldsymbol{y}_{fb}(t)\right) \geq 0 \quad \text{(path constraints)} \quad (5)$$

$$\mathbf{l}\left(\boldsymbol{x}(0), \boldsymbol{x}(t_f)\right) = 0 \quad \text{(boundary conditions)} \quad (6)$$

with the deterministic controls consisting of the feedforward excitations $\boldsymbol{e}_{ff}(t)$and the time-varying linear feedback gain matrix $\boldsymbol{K}(t)$, $\boldsymbol{x}(t)$ the stochastic states, $\boldsymbol{y}_{fb}(t)$ is the stochastic feedback signal,

$\boldsymbol{e}(t)$ are the total muscle excitations (combination of feedforward and feedback), $\boldsymbol{w}_{sys}$ **is** Gaussian system noise (e.g. motor noise), and $\boldsymbol{w}_{policy}$ is Gaussian noise corrupting the policy (e.g. sensory noise). The system and policy noise inject uncertainty into the simulations, resulting in stochastic state variables. Consequently, all variables that depend on the state are stochastic as well. The controls, $\boldsymbol{e}_{ff}(t)$ and $\boldsymbol{K}(t)$, are deterministic variables.

Although the noise sources are Gaussian, the distribution of the stochastic variables are generally non-Gaussian due to nonlinearities in the dynamics. In the proposed approach, we approximate the state distribution as Gaussian. Consequently, we can describe the distribution of the state trajectory by the mean state trajectory ($\boldsymbol{x}_{mean}(t)$) and the state covariance trajectory ($\boldsymbol{P}(t)$). The dynamics of the mean state trajectory are approximated by the deterministic ($\boldsymbol{w} = [\boldsymbol{w}_{sys}, \boldsymbol{w}_{policy}] = 0$) version of the system dynamics $\boldsymbol{f}(\cdot)$. The propagation of the state covariance is approximated using the continuous Lyapunov differential equations [6], [7], which assume local invariance of the system dynamics around the mean trajectory:

$$\dot{\boldsymbol{P}}(t) = \boldsymbol{A}(t)\boldsymbol{P}(t) + \boldsymbol{P}(t)\boldsymbol{A}(t)^{\boldsymbol{T}} + \boldsymbol{C}(t)\Sigma'_w\boldsymbol{C}(t) \tag{7}$$

$$\boldsymbol{A}(t) = \frac{\partial \boldsymbol{f}}{\partial \boldsymbol{x}}\left(\boldsymbol{x}(\boldsymbol{t}), \boldsymbol{e}_{ff}(t), \boldsymbol{K}(t), \boldsymbol{w}\right) \tag{8}$$

$$\boldsymbol{C}(t) = \frac{\partial \boldsymbol{f}}{\partial \boldsymbol{w}}\left(\boldsymbol{x}(\boldsymbol{t}), \boldsymbol{e}_{ff}(t), \boldsymbol{K}(t), \boldsymbol{w}\right) \tag{9}$$

with Σ'_w the covariance matrix describing Gaussian noise sources. The propagation of the state covariance matrix is as in an Extended Kalman Filter [8].

The propagation of $\boldsymbol{P}(t)$ can be interpreted as the change in uncertainty due to the dynamics ($\boldsymbol{A}(t)\boldsymbol{P}(t) + \boldsymbol{P}(t)\boldsymbol{A}(t)^{\boldsymbol{T}}$) and the injection of uncertainty by the noise sources ($\boldsymbol{C}(t)\Sigma'_w\boldsymbol{C}(t)$). The dynamics can either act to dissipate uncertainty or to increase uncertainty depending on the system.

Based on the distribution of the stochastic variables, the constraints can be robustified as well. To this aim, the distribution of the constraint equations is approximated based on the assumption of a Gaussian state distribution. Hence, the constraint standard deviation is $\sqrt{\frac{\partial g_i}{\partial x}\boldsymbol{P}(t)\frac{\partial g_i}{\partial x}^T}$

The constraints are then reformulated as follows:

$$g_i\left(\boldsymbol{x}(t), \boldsymbol{e}_{ff}(t), \boldsymbol{K}(t), \boldsymbol{y}_{fb}(t)\right) + \gamma\sqrt{\frac{\partial g_i}{\partial \boldsymbol{x}}\boldsymbol{P}(t)\frac{\partial g_i}{\partial \boldsymbol{x}}^T} \geq 0 \tag{10}$$

with γ a parameter that defines the chance that the constraint is fulfilled. Due to the use of a Gaussian state distribution, even solutions far away of the mean state have a finite chance of occurring and therefore it will not be possible to impose the constraints for the entire state distribution.

Positive definiteness preserving discretization

The state covariance matrix $\boldsymbol{P}(t)$ is positive-definite by definition. However, numerical integration can destroy this property due to the accumulation of integration errors. Gillis and Diehl noted and addressed this issue [9]; they propose a positive-definiteness preserving discretization of the Lyapunov differential equation as explained below. The mathematical proof can be found in [9], [10].

If direct collocation is applied to transcribe the optimal control problem, the dynamics can be discretized into a set of constraint functions F and G that describe the numerical integration:

$$x_{k+1} = F(z_k) \tag{11}$$

$$0 = G(\delta;\ x_k, z_k, e_k, w_k) \tag{12}$$

with z_k auxiliary variables defining the state at the collocation points within each integration interval and δ the integration interval. It is shown in [10], [11] that given the discretized dynamics in the above form, the continuous propagation of the state covariance matrix can be discretized into the following form that guarantees the preservation of positive definiteness:

$$P_{k+1} = M_k \left(\frac{\partial G}{\partial x} P_k \frac{\partial G}{\partial x}^T + \frac{\partial G}{\partial w} \Sigma_w \frac{\partial G}{\partial w}^T \right) M_k^T \tag{13}$$

$$0 = \frac{\partial F}{\partial z}^T - \frac{\partial G}{\partial z}^T M_k^T \tag{14}$$

For example, in the case of a trapezoidal integration scheme the discretized dynamics (eq. (11) and eq. (12)) of a system with continuous dynamics in the general form $\dot{x} = \mathrm{f}(x, u, \mathrm{w})$ become:

$$x_{k+1} = z_k \tag{15}$$

$$0 = z_k - \left(x_k + \frac{\delta}{2}(\dot{x}_k + \dot{x}_{k+1}) \right) \Leftrightarrow$$

$$0 = z_k - \left(x_k + \frac{\delta}{2}\big(f(x_k, u_k, w) + f(z_k, u_{k+1}, w)\big) \right) \tag{16}$$

Combination with implicit formulation of system dynamics

Formulating the dynamic equations implicitly, $f_{imp}(x, u, \dot{x}, w) = 0$, rather than explicitly, $\dot{x} = f_{exp}(x, u, w)$ has been shown to improve the efficiency of deterministic optimal control simulations of movement [12]. This approach avoids the inversion of near-singular matrices (e.g. the mass matrix in multibody systems with bodies of different mass, length and inertia), which is numerically an unstable operation and can become an issue for underlying NLP solvers. In practice, when direct collocation is applied and the dynamics are formulated implicitly, one can augment the variable space with ‘slack controls’ ($u_{\dot{x}}$) that represent the state derivatives and impose the system dynamics in an implicit manner: $f_{imp}(x, u, u_{\dot{x}}, w) = 0$. When using an implicit formulation and a trapezoidal integration scheme, the discretized dynamics become:

$$x_{k+1} = z_k \tag{17}$$

$$0 = z_k - (x_k + \frac{\delta}{2}\big(u_{\dot{x},k} + u_{\dot{x},k+1}\big)) \tag{18}$$

$$f_{imp}(x, u, u_{\dot{x}}, w) = 0 \tag{19}$$

To formulate the propagation of the state covariance matrix, the sensitivity of the state derivatives with respect to the states and noise sources is required (eq. 8 and eq. 9). When the dynamics are formulated implicitly these expressions are not readily available. To conserve the possibility to formulate the system dynamics implicitly it is required to augment the variable space once again with ‘slack controls’ that represent the derivatives of the state derivatives with respect to the state ($u_{\partial\dot{x}\partial x}$) and the noise sources ($u_{\partial\dot{x}\partial w}$). We determine those through the following (implicit) constraints that follows from taking the partial derivatives to the state x and the noise sources w of eq. 19 and applying the chain rule:

$$\frac{\partial f_{imp}(x, u, u_{\dot{x}}, w)}{\partial x} + \frac{\partial f_{imp}(x, u, u_{\dot{x}}, w)}{\partial u_{\dot{x}}} u_{\partial\dot{x}\partial x} = 0 \tag{20}$$

$$\frac{\partial f_{imp}(x, u, u_{\dot{x}}, w)}{\partial w} + \frac{\partial f_{imp}(x, u, u_{\dot{x}}, w)}{\partial u_{\dot{x}}} u_{\partial\dot{x}\partial w} = 0 \tag{21}$$

Eq. 20 and eq. 21 can then be combined with the positive definiteness preserving Lyapunov discretization. In case of the trapezoidal integration scheme we obtain:

$$\frac{\partial G}{\partial x} = -I - \frac{\delta}{2} u_{\partial \dot{x} \partial x,k} \quad (22)$$

$$\frac{\partial G}{\partial z} = I - \frac{\delta}{2} u_{\partial \dot{x} \partial x,k+1} \quad (23)$$

$$\frac{\partial F}{\partial z} = I \quad (24)$$

$$\frac{\partial G}{\partial w} = -\frac{\delta}{2} (u_{\partial \dot{x} \partial w,k} + u_{\partial \dot{x} \partial w,k+1}) \quad (25)$$

Approximate stochastic optimal control framework

Here, we describe the approximate stochastic optimal control problem for the class of problems we are solving in this study. Given the implicit stochastic dynamics: $\boldsymbol{f}(\boldsymbol{x}(t), \dot{\boldsymbol{x}}(t), \boldsymbol{e}_{ff}(t), \boldsymbol{K}(t), \boldsymbol{w}_{sys}, \boldsymbol{w}_{policy}) = 0$, with a control policy that is defined within the dynamics wherethe controls::

$$\boldsymbol{e}(t) = \boldsymbol{e}_{ff}(t) + \boldsymbol{K}(t) * (\boldsymbol{y}_{fb}(\boldsymbol{x}(t)) + \boldsymbol{w}_{policy}) + \boldsymbol{w}_{\boldsymbol{sys}} \quad (26)$$

We search the deterministic controls, $\boldsymbol{e}_{ff}(t)$ and $\boldsymbol{K}(t)$, and reference trajectory $\boldsymbol{p}_{ref}(t)$ that minimize the expected control effort $\mathrm{J} = \mathrm{E}[\int \boldsymbol{e}^T(t)\boldsymbol{e}(t)dt]$ under some constraints that specify the simulated task.

Using a trapezoidal integration scheme and the theory introduced in previous sections we can write the approximate discretized stochastic optimal control as follows when discretizing the time horizon [0 T] into N intervals of length δ:

$$\min_{\boldsymbol{e}_{ff}, \boldsymbol{K}} \quad J = \sum_k \boldsymbol{e}_{ff,k}^T \boldsymbol{e}_{ff,k} + \sum_k tr(\boldsymbol{K}_k Var(\boldsymbol{y}_{fb,k}) \boldsymbol{K}_k^T) + \sum_k tr(\boldsymbol{K}_k \Sigma_{w_{policy}} \boldsymbol{K}_k^T) \quad (27)$$

$s.t.$

$$\boldsymbol{x}_{mean,\mathrm{k+1}} - \left(\boldsymbol{x}_{mean,\mathrm{k}} + \frac{\delta}{2} (\boldsymbol{u}_{\dot{x},k} + \boldsymbol{u}_{\dot{x},k+1}) \right) = 0 \quad (28)$$

$$\boldsymbol{P}_{k+1} = \boldsymbol{M}_k \left(\left(\frac{\partial \boldsymbol{G}}{\partial \boldsymbol{x}} \right)_k \boldsymbol{P}_k \left(\frac{\partial \boldsymbol{G}}{\partial \boldsymbol{x}} \right)_k^T + \left(\frac{\partial \boldsymbol{G}}{\partial \boldsymbol{w}} \right)_k \Sigma_w \left(\frac{\partial \boldsymbol{G}}{\partial \boldsymbol{w}} \right)_k^T \right) \boldsymbol{M}_k^T \quad (29)$$

$$\boldsymbol{f}_{imp}(\boldsymbol{x}_{mean,k}, \boldsymbol{e}_{ff,k}, \boldsymbol{K}_k, \boldsymbol{u}_{\dot{x},k}) = 0 \quad (30)$$

$$\frac{\partial \boldsymbol{f}_{imp}(\boldsymbol{x}_{mean,k}, \boldsymbol{e}_{ff,k}, \boldsymbol{K}_k, \boldsymbol{u}_{\dot{x},k}, \boldsymbol{w})}{\partial \boldsymbol{x}} + \frac{\partial \boldsymbol{f}_{imp}(\boldsymbol{x}_{mean,k}, \boldsymbol{e}_{ff,k}, \boldsymbol{K}_k, \boldsymbol{u}_{\dot{x},k}, \boldsymbol{w})}{\partial \boldsymbol{u}_{\dot{x}}} \boldsymbol{u}_{\partial \dot{x} \partial x,k} = 0 \quad (31)$$

$$\frac{\partial \boldsymbol{f}_{imp}(\boldsymbol{x}_{mean,k}, \boldsymbol{e}_{ff,k}, \boldsymbol{K}_k, \boldsymbol{u}_{\dot{x},k}, \boldsymbol{w})}{\partial \boldsymbol{w}} + \frac{\partial \boldsymbol{f}_{imp}(\boldsymbol{x}_{mean,k}, \boldsymbol{e}_{ff,k}, \boldsymbol{K}_k, \boldsymbol{u}_{\dot{x},k}, \boldsymbol{w})}{\partial \boldsymbol{u}_{\dot{x}}} \boldsymbol{u}_{\partial \dot{x} \partial w,k} = 0 \quad (32)$$

$$\left(\frac{\partial \boldsymbol{G}}{\partial \boldsymbol{x}} \right)_k = -I - \frac{\delta}{2} \boldsymbol{u}_{\partial \dot{x} \partial x,k}; \left(\frac{\partial \boldsymbol{G}}{\partial \boldsymbol{z}} \right)_k = I - \frac{\delta}{2} \boldsymbol{u}_{\partial \dot{x} \partial x,k+1} \quad (33)$$

$$\left(\frac{\partial \boldsymbol{F}}{\partial \boldsymbol{z}} \right)_k = I; \left(\frac{\partial \boldsymbol{G}}{\partial \boldsymbol{w}} \right)_k = -\frac{\delta}{2} (\boldsymbol{u}_{\partial \dot{x} \partial w,k} + \boldsymbol{u}_{\partial \dot{x} \partial w,k+1}) \quad (34)$$

$$0 = \left(\frac{\partial \boldsymbol{F}}{\partial \boldsymbol{z}} \right)_k^T - \left(\frac{\partial \boldsymbol{G}}{\partial \boldsymbol{z}} \right)_k^T \boldsymbol{M}_k^T \quad (35)$$

$$g_i(\boldsymbol{x}_{mean,k}, \boldsymbol{e}_{ff,k}, \boldsymbol{K}_k, \boldsymbol{p}_{ref,k}) + \gamma_i \sqrt{\frac{\partial g_i}{\partial x} \boldsymbol{P}(t) \frac{\partial g_i}{\partial x}^T} \geq 0 \quad with\ i = 1 \dots n_g. \quad (36)$$

With $\boldsymbol{M}_k$ a helper variable introduced to avoid the inversion of the matrix $\left(\frac{\partial G}{\partial z}\right)_k^T$. Note further that through linearization we can write:

$$Var(\boldsymbol{y}_{fb,k}) = \frac{\partial \boldsymbol{h}(\boldsymbol{x}_{mean,k'})}{\partial \boldsymbol{x}} \boldsymbol{P}_k \frac{\partial \boldsymbol{h}(\boldsymbol{x}_{mean,k'})}{\partial \boldsymbol{x}}^T \tag{37}$$

Equation (27) can be derived from equation (1) since deterministic variables are independent from stochastic variables ($E[e_{ff} \cdot e_{fb}] = E[e_{ff}] \cdot E[e_{fb}]$), the expected value of the feedback signal is zero ($E[e_{fb}] = 0$), all noise sources are independent, and the variance of deterministic variables, such as the feedforward muscle excitations e_{ff}, is zero ($Var(e_{ff}) = 0$):

$$E[e^2] = E[e_{ff}^2] + E[e_{fb}^2] + 2 \cdot E[e_{ff} \cdot e_{fb}]$$

$$E[e^2] = E[e_{ff}]^2 + Var(e_{ff}) + E[e_{fb}]^2 + Var(e_{fb}) + 2 \cdot E[e_{ff}] \cdot E[e_{fb}]$$

$$E[e^2] = E[e_{ff}]^2 + 0 + 0 + Var(e_{fb})$$

$$E[e^2] = E[e_{ff}]^2 + Var(K \cdot y_{fb}) + Var(K \cdot w_s) + Covar(K \cdot y_{fb}, K \cdot w_s)$$

$$E[e^2] = E[e_{ff}]^2 + tr(K \cdot Var(y_{fb}) \cdot K') + tr(K \cdot \Sigma_{w_s} \cdot K') + 0$$

where $Covar(K \cdot y_{fb}, K \cdot w_s) = 0$ as the noise sources are independent.

Notes on integration schemes

When the system dynamics are not known a priori, because the feedback gains that determine the closed-loop dynamics are optimization variables, we could end up in a situation where the propagation of the covariance is inaccurate depending on the chosen discretization scheme. Backward and forward Euler integrations schemes are problematic, whereas a trapezoidal integration scheme is more reliable.

Why a forward Euler scheme is problematic for the propagation of covariance

In the case of using a forward Euler discretization scheme, eq. 11 and eq. 12 become:

$$x_{k+1} = z_k \tag{38}$$
$$0 = z_k - (x_k + \delta u_{\dot{x},k}) \tag{39}$$

This leads to the following expressions for the positive definiteness preserving Lyapunov discretization scheme:

$$\frac{\partial G}{\partial x} = -I - \delta u_{\partial \dot{x} \partial x,k}; \tag{40}$$
$$\frac{\partial G}{\partial z} = I; \ \frac{\partial F}{\partial z} = I; \tag{41}$$
$$\frac{\partial G}{\partial w} = -\delta u_{\partial \dot{x} \partial w,k} \tag{42}$$

We thus obtain:

$$P_{k+1} = \left((I + \delta u_{\partial \dot{x} \partial x,k}) P_k (I + \delta u_{\partial \dot{x} \partial x,k})^T + \ \delta u_{\partial \dot{x} \partial w,k} \Sigma_w \delta u_{\partial \dot{x} \partial w,k}{}^T\right) \tag{43}$$

To demonstrate why a backward Euler scheme might be problematic, we only consider the first in eq. 44 that describes how the covariance matrix changes due to the dynamics. For this example, let us consider that the dynamics describes exponential growth/decay: $\dot{x} = \alpha \cdot x$. If $\alpha < 0$ initial variance of the state x decreases over time, with a faster decrease of uncertainty with increasing absolute value of

α. If $\alpha > 0$ initial variance of the state x increases over time, with a faster increase of uncertainty with increasing absolute value of α.

In this case the discrete covariance propagation equation becomes:

$$P_{k+1} = (1 + \delta \cdot \alpha)^2 P_k \tag{44}$$

We observe the following problems.

(1) The uncertainty can be completely eliminated from the system by choosing $\alpha = -1/\delta$. Although a negative alpha does stabilize the dynamics and uncertainty would asymptotically disappear over time, this could result in strong over-estimation of the dissipative features of the dynamics.
(2) When $\alpha \ll -1/\delta$ and thus we have a strongly dissipative system, uncertainty will accumulate strongly. The discrete dynamics do not capture the dissipative feature of the system dynamics in this case. One could argue that δ must be chose smaller, but if α is an optimization variable, it cannot be verified in advance whether δ is small enough.

If for example the objective is to minimize the uncertainty in the system, the optimization will select $\alpha = -1/\delta$ while an $\alpha \ll -1/\delta$ would in reality be a better solution.

Why a backward Euler scheme is problematic for the propagation of covariance

In the case of using a backward Euler discretization scheme, eq. 11 and eq. 12 become (appreciate the difference with the forward Euler scheme):

$$x_{k+1} = z_k \tag{45}$$

$$0 = z_k - (x_k + \delta u_{\dot{x},k+1}) \tag{46}$$

This leads to the following equation for the discrete covariance propagation:

$$\frac{\partial G}{\partial x} = -I; \tag{47}$$

$$\frac{\partial G}{\partial z} = I - \delta u_{\partial\dot{x}\partial x,k}; \frac{\partial F}{\partial z} = I; \tag{48}$$

$$\frac{\partial G}{\partial w} = -\delta u_{\partial\dot{x}\partial w,k+1} \tag{49}$$

We thus obtain:

$$P_{k+1} = (I - \delta u_{\partial\dot{x}\partial x,k})^{-1}\left(P_k + \delta u_{\partial\dot{x}\partial w,k+1}\Sigma_w \delta u_{\partial\dot{x}\partial w,k+1}{}^T\right)((I - \delta u_{\partial\dot{x}\partial x,k})^{-1})^T \tag{50}$$

Again, we ignore the term due to noise and only consider the dynamics term. Let us consider the same dynamics as in the previous example: $\dot{x} = \alpha \cdot x$. In this case we find:

$$P_{k+1} = \frac{1}{(1 - \delta \cdot \alpha)^2} P_k \tag{51}$$

In contrast to the forward Euler scheme, there is no finite α that would eliminate all uncertainty in a single time step. If we choose $\alpha < 0$, we will dissipate uncertainty from the system as is expected. The larger the time step δ, the more we will underestimate how much uncertainty is dissipated from the system. With smaller step size we approach the true value. If we choose $1 > \delta \cdot \alpha > 0$ it is clear that uncertainty will accumulate, which makes sense. However, we observe again two issues:

(1) When choosing $\delta \cdot \alpha = 1$ the uncertainty will become infinite at the next time-step. This can become an issue during an iterative optimization process where an iteration might bump into values that are infinite.

(2) When $\delta \cdot \alpha > 2$, the system appears to dissipate uncertainty while in reality it should be accumulating uncertainty.

Trapezoidal scheme to solve these integrator stability issues

In case of a trapezoidal scheme, the same example dynamics $\dot{x} = \alpha \cdot x$ results in the following discrete covariance propagation equation:

$$P_{k+1} = \frac{(1 + \frac{\delta}{2} \cdot \alpha)^2}{(1 - \frac{\delta}{2} \cdot \alpha)^2} P_k \tag{52}$$

The behavior (accumulative/dissipative) of the system integrated by this integrator is correct for all values of α.

If $\alpha \gg 0$ we still have an accumulation of uncertainty as the asymptote lies at 1 for $\frac{\delta}{2} \cdot \alpha = +inf$.

If $\alpha < 0$ we have a dissipative system.

There is an asymptote where uncertainty accumulates infinitely quick if $\frac{\delta}{2} \cdot \alpha = 1$ which can still give us an issue during optimization. However, current gradient-based optimization algorithms for solving nonlinear programs (IPOPT [13], SNOPT [14]) have heuristics implemented that make them relatively robust to such problems.

References

[1] C. Harris and D. Wolpert, "Signal-dependent Noise determines Motor Planning," *Nature*, vol. 394, pp. 780–784, 1998, Accessed: Aug. 31, 2017. [Online]. Available: http://wexler.free.fr/library/files/harris (1998) signal-dependent noise determines motor planning.pdf.

[2] T. Flash and N. Hogan, "The coordination of arm movements: An experimentally confirmed mathematical model," *J. Neurosci.*, vol. 5, no. 7, pp. 1688–1703, 1985, doi: 10.1523/jneurosci.05-07-01688.1985.

[3] F. Crevecoeur, S. H. Scott, and T. Cluff, "Robust Control in Human Reaching Movements: A Model-Free Strategy to Compensate for Unpredictable Disturbances," *J. Neurosci.*, vol. 39, no. 41, pp. 8135–8148, Oct. 2019, doi: 10.1523/JNEUROSCI.0770-19.2019.

[4] H.-K. Kim and Y. Zhang, "Estimation of lumbar spinal loading and trunk muscle forces during asymmetric lifting tasks: application of whole-body musculoskeletal modelling in OpenSim," *http://dx.doi.org/10.1080/00140139.2016.1191679*, vol. 60, no. 4, pp. 563–576, Apr. 2016, doi: 10.1080/00140139.2016.1191679.

[5] A. van der Have, S. Van Rossom, and I. Jonkers, "Squat Lifting Imposes Higher Peak Joint and Muscle Loading Compared to Stoop Lifting," *Appl. Sci. 2019, Vol. 9, Page 3794*, vol. 9, no. 18, p. 3794, Sep. 2019, doi: 10.3390/APP9183794.

[6] B. Houska, "Robust optimization of dynamic systems," 2011, Accessed: Sep. 04, 2020. [Online]. Available: http://dl.sanaye20.ir/RobustOptimazation.pdf.

[7] B. Houska and M. Diehl, "Robustness and stability optimization of power generating kite systems in a periodic pumping mode," in *Proceedings of the IEEE International Conference on Control Applications*, 2010, pp. 2172–2177, doi: 10.1109/CCA.2010.5611288.

[8] S. J. Julier, J. K. Uhlmann, and H. F. Durrant-Whyte, "New approach for filtering nonlinear

systems," in *Proceedings of the American Control Conference*, 1995, vol. 3, pp. 1628–1632, doi: 10.1109/acc.1995.529783.

[9] J. Gillis and M. Diehl, "A positive definiteness preserving discretization method for lyapunov differential equations," in *Proceedings of the IEEE Conference on Decision and Control*, 2013, pp. 7759–7764, doi: 10.1109/CDC.2013.6761121.

[10] J. Gillis, "Practical methods for approximate robust periodic optimal control of nonlinear mechanical systems," no. March, 2015.

[11] J. Gillis and M. Diehl, "A positive definiteness preserving discretization method for lyapunov differential equations," *Proc. IEEE Conf. Decis. Control*, pp. 7759–7764, 2013, doi: 10.1109/CDC.2013.6761121.

[12] A. J. Van Den Bogert, D. Blana, and D. Heinrich, "Implicit methods for efficient musculoskeletal simulation and optimal control," in *Procedia IUTAM*, 2011, vol. 2, no. 2011, pp. 297–316, doi: 10.1016/j.piutam.2011.04.027.

[13] A. Wächter and L. T. Biegler, "On the implementation of an interior-point filter line-search algorithm for large-scale nonlinear programming," *Math. Program.*, vol. 106, no. 1, pp. 25–57, 2006, doi: 10.1007/s10107-004-0559-y.

[14] P. E. Gill, W. Murray, and M. A. Saunders, "SNOPT: An SQP Algorithm for Large-Scale Constrained Optimization," *http://dx.doi.org/10.1137/S0036144504446096*, vol. 47, no. 1, pp. 99–131, Aug. 2006, doi: 10.1137/S0036144504446096.
