# Supplementary material for: An approximate stochastic optimal control framework to simulate nonlinear neuro-musculoskeletal models in the presence of noise
Source: PLoS Comput Biol. 2022 Jun 8;18(6):e1009338. doi: 10.1371/journal.pcbi.1009338 (PMC9176817; doi:10.1371/journal.pcbi.1009338)
Supplement: Attachment — Submitted filename: PCOMPBIOL-D-21-01394_comments.docx [file pcbi.1009338.s002.docx]

This manuscript presents a novel method to perform predictive movement simulations using neuromusculoskeletal system models that include random perturbations, motor noise, and sensory noise. The method is applied to standing, where the effect of perturbations, muscle properties, and sensory loss on the optimal neural control parameters is predicted. The method is also applied to reaching movements, to predict the optimal trajectories and the corresponding feedback-feedforward control strategies.

This is a very exciting manuscript that addresses an important problem. Experiments have suggested that movement trajectories and muscle control are affected by noise and perturbations. Traditional optimal feedback control theories for linear systems are unable to explain and predict this. There has not been an effective method for systems with nonlinearities such as occur in muscle mechanics. This paper could have a large impact on the field and encourage new applications of this approach. Unlike previous work, this new approach has the potential to be scalable to larger musculoskeletal systems and more complex tasks.

However, my enthusiasm is diminished because the authors try to do too much, which has led to some significant weaknesses, errors, and unnecessary clutter. This may leave the reader unconvinced and lost. It is hard to see the great fundamental contribution when distracted by these weaknesses.

I would love to see a thorough rewrite of the paper, and perhaps focus on the essentials, without trying to simulate vestibular loss or short-range muscle stiffness, or too many separate noise contributions. The main goal should be to present the method and convince the reader that it works, and that the predictions make sense and agree with experiments.

Specific comments follow below.

1. I will start with the main weakness which almost made me ignore everything else. The cost function (9) is constructed in a manner that favors an equal distribution of effort between feedforward and feedback. For example, if a static posture requires a constant 50% activation level in just one muscle, it could be generated with feedforward or feedback, or any combination of both. The only difference would be the stability of the system which only plays a role in the stochastic case. However, with this cost function, the cost would be 0.25 (0.5^2) when using only feedback, and 0.25 when only using feedforward, but only 0.125 (0.25^2 + 0.25^2) when using equal amounts of feedforward and feedback. Hence that solution would be optimal, and this has nothing to do with stochastic dynamics. The cost function should clearly be related to total activation squared, not separated into feedback and feedforward components. The work should be repeated with the proper cost function to avoid favoring certain solutions.
2. The Introduction may benefit from reminding readers that linearization will produce the same optimal control law, and the same optimal trajectory, regardless of the amount of noise in the system (due to the Certainty Equivalence principle). Linearization would never predict that trajectories and control laws depend on noise level, as suggested by experiments and by the authors’ novel optimization method. A good example of linearization is: He, Levine & Loeb, IEEE Trans Autom Control 1991.
3. Line 198, “delayed proprioceptive and vestibular cues”. As far as I can tell, the feedback control (eq. 14 in Methods) does not have time delay.
4. Line 228, “moderate levels”. If I read the graph correctly, the CCI is 2% which seems low, rather than moderate.
5. Line 259. I feel that the work would be much stronger if a more conventional Hill model was used, with a series elasticity rather than a rigid tendon, and without the added short range stiffness. A nonlinear series elasticity would create a force-dependent stiffness, and it would be truly a short range stiffness because the stiffness decreases as soon as the contractile element “catches up” with an external stretch (e.g. GK Cole et al., J Biomech 1996). Series elasticity can be seen as the combined elastic properties of tendon, aponeurosis, and cross-bridges. The SRS model may give the impression of an artificial “trick” to introduce a benefit of co-contraction. As modeled in eq. (10), “short range” is a misnomer. The stiffness is only dependent on activation and not on the amount of stretch. It would be best to eliminate this controversy and show that predictions can be obtained with a conventional Hill model. Otherwise, readers may think the method is not applicable to the usual musculoskeletal models.
6. Methods. The problem formulation (eqs. 1-8) is very elegant and presented very clearly. Especially the incorporation of the state covariance into the path constraint is a great idea, with a meaningful parameter to specify a required probability of success.
7. Supplementary material. Please delete any co-author comments that still appear in the margin.
8. Methods, equation (13). The equation of motion would be more clear if formulated in terms of absolute angle (relative to gravity) rather than ankle angle (relative to platform). As presented, the equation of motion is in a non-inertial reference frame, since qA is defined relative to the platform. With absolute angle, the w_SS,q and w_SS,qdd would not be needed here. The platform rotation would then have to accounted for in the relationship between absolute angle and muscle length, where the platform angle must be added to the absolute angle, to obtain ankle angle. I don’t think it would affect the nature of the problem (they are all noise terms) but it would be cleaner not to have to specify the angular acceleration noise. But also see comment 15.
9. Methods, equation (13). The platform translation term in the equation of motion is incorrect, sin(qA) should be changed to cos(qA). As written, this term represents the effect of a vertical platform acceleration. Hopefully this is a typing error and the correct model was used in the computational work.
10. Line 585. When following my suggestion in comment 8, angular velocity of the platform would still need to be specified because it affects contractile element velocity. However, this would no longer be needed when series elasticity is used in the muscle model as suggested in comment 5. Only platform angle would be needed.
11. Line 585. If angle, angular velocity, and angular acceleration are all included in the model (which hopefully is not the case, see above), I think they can’t all be modeled independently as white noise. If the angular acceleration is white noise, the angular velocity would be the integral of white noise, i.e. Brownian motion which is not white noise.
12. Methods. The noise terms are printed in red font, but you should probably not assume that this can be rendered like that in the published paper.
13. Methods, equation (9). The final term should have subscript TA rather than SOL. But see also comment 1.
14. Methods, equation (10). It is odd that the short range stiffness is only dependent on the baseline activation. If this is a mechanical muscle property, it can’t depend on how the activation is generated by neural circuits. It can only depend on the (total) activation. Hopefully this will be a moot point when comment 5 is addressed.
15. Methods, equation (14). If q_A is the ankle angle, the w_SS terms should not be in the proprioception model. However if my suggestion is followed and the equations of motion are formulated in absolute angle, the platform motion is correctly represented here.
16. Methods, equation (14). The feedback model has redundant parameters, and the optimization problem may not have a unique solution. For instance, e_SOL,fb = K11*(qA + w_p,q) + K13*(qA + w_SS,q + w_v,q) + ... = (K11+K13)*qA + ... Therefore, K11 and K13 cannot be optimized indepedently, only their sum is relevant. This suggest that the problem must be simplified, which will also make the work more convincing. Similarly, some noise terms are mostly (sometimes exactly) additive, so only their sum is relevant. It may be more convincing to only have the noise terms that uniquely influence the solution. This will make the work appear less cluttered, and prevent the impression that results are dependent in numerous, somewhat arbitrary, assumptions about noise levels.
17. Line 614. Co-contraction index (CCI) was defined as the activation of the largest muscle. This does not resemble any other definitions of CCI that have been used in the literature. Always, the CCI is calculated from both activations: the agonist and the antagonist. And the CCI equation should always be symmetrical: if the two activations are switched, the CCI should remain the same. This definition only uses one muscle’s activation and should therefore not be named “co-contraction”. Consider using a more standard definition of CCI.
18. The formatting of the Tables should be improved. All muscles should have a separate column. Sometimes columns are broken in two parts that are not vertically aligned.
19. Table 2 has a note on the units of noise variance. Indeed the units are correct, but the explanation does not seem very helpful. Hopefully this can be improved. Would it be better to say: (m/s^2)^2 / Hz, and mention that this is represents the power spectral density? Readers may be more familiar with continuous-time noise described in the frequency domain.
20. Line 684 refers to Figure 2, but the model is shown in Figure 3. It is odd that results (Figure 2) are presented before the model.
21. Musculoskeletal models for 2-DOF reaching will typically have six muscles, including two biarticular muscles. Such models are easily replicated from the literature or from Opensim. Is there a particular reason for not doing this?
