# Supplementary material for: An approximate stochastic optimal control framework to simulate nonlinear neuro-musculoskeletal models in the presence of noise
Source: PLoS Comput Biol. 2022 Jun 8;18(6):e1009338. doi: 10.1371/journal.pcbi.1009338 (PMC9176817; doi:10.1371/journal.pcbi.1009338)
Supplement: Attachment — Submitted filename: ResponseToReviewers.docx [file pcbi.1009338.s003.docx]

REVIEW SOC PAPER

**REVIEWER 1**

This article presents an approximate stochastic optimal control framework for investigating feedforward and feedback control in nonlinear neuromusculoskeletal systems with sensorimotor noise. Several simulations are performed for standing and reaching tasks with perturbations and the results are compared to previous experimental data.

Overall, the paper is well-written and develops interesting ideas about the modeling of sensorimotor control. It makes original contributions by applying the framework to standing and using state-of-the-art neuromusculoskeletal models for instance. However, several points need to be addressed to complete the literature, improve the consistency of the modeling work, and clarify its implications for the neural control of movement.

Thanks for the detailed review. It really helped to clarify some things that were not communicated clearly and made the paper more comprehensive and accurate. We tried to include the references the reviewer noted and address these both in the introduction and discussion. A major change with respect to the previous submission is that we updated the musculoskeletal model for the reaching task to include 6 muscles (4 uniarticular and 2 biarticular).

Main comments

First, it must be noted that recent papers developed and applied a very similar approximate stochastic optimal control framework to arm reaching with noisy nonlinear musculoskeletal models (see references 1-3 below). Currently, these papers are not referenced. Due to the similarity with these approaches, the authors should clearly stress and discuss the novelty of their results with respect to these studies as well as revise several statements throughout the paper accordingly.

Thanks for pointing us to these references. They are indeed relevant to our work. We now cite these papers and explicitly explain how our approach is similar but goes beyond this earlier work.

Specifically, the two earlier papers consider only feedforward control, whereas our work simultaneously optimizes feedforward and feedback control.

In the latest paper, feedback and feedforward were solved for sequentially. Separately optimizing feedforward and feedback control will generally lead to suboptimal control. For example, optimizing stability in the absence of feedback control might require high levels of muscle co-contraction. This solution might be suboptimal with respect to effort minimization if feedback control is available as well. We therefore think it is important to simultaneously solve for feedforward and feedback control parameters that optimize performance.

We added a paragraph to the introduction:

“Berret et al. [33] have applied this approach to simulations of reaching [35] [36]. However, they only considered feedforward control [35] or added feedback control to the pre-computed optimal feedforward control in a post-processing step using the LQG framework [35]. This does not seem to yield an optimal solution as the during the optimization of the feedforward control policy, the presence of feedback is neglected. It is therefore likely that co-optimizing feedforward and feedback contributions might result in better and more realistic control policies.”

In the discussion we come back to the work as well:

“Muscle co-contraction has been observed experimentally during reaching [87], especially in a divergent force field [61], [63], [64], but was not predicted by our stochastic optimal control simulations. Berret et al. only considered feedforward control in their simulations of reaching and found higher levels of co-contraction [35] as in the absence of feedback control, co-contraction is the only mechanism to increase joint impedance and hence robustness against perturbations during reaching. Our simulations suggest that muscle co-contraction is an energetically costly strategy when feedback is present resulting in very low simulated levels of co-contraction….”

Second, there are some inconsistencies in the modeling work. The general frameworks of the main text and of the SI text do not correspond exactly, and some discrepancy between the general framework and the specific examples can be noted. For instance, in the standing task, the feedforward control acts at the level of muscle activations (a_base) but both the feedforward and feedback control terms should act at the level of muscle excitations if we refer to the general framework (as done for reaching). Similarly, motor noise should be introduced at the level of muscle excitations if we refer to the general framework described in SI, but both in the standing and reaching tasks it is introduced at the level of muscle force/torque (yet it fits with the framework described in the main text, L. 167).

Thanks for pointing out these discrepancies. We updated the general formulation so that it is really general, leaving open the possibility how noise is coming into the simulations as a modelling choice. We describe how we implemented how noise is “entering” our simulations in the methods section.

We chose in the general formulation for noise that can be added at the level of the system/musculoskeletal dynamics (system noise) or as a part of the control policy (policy noise):

“We formulate predictive simulations of human movement as stochastic optimal control problems of the following form:

| $\min_{e_{ff}\left( t \right),K\left( t \right),p_{ref}\left( t \right)}$ | | $J=E[\int e^{T}\left( t \right)e\left( t \right)dt]$. | | (1) |
| --- | --- | --- | --- | --- |
| $subject to$ | $\dot{x}\left( t \right)=f\left( x\left( t \right),e\left( t \right),w_{sys} \right)$  $e\left( t \right)=e_{ff}\left( t \right) +K\left( t \right)*y_{fb}\left( x\left( t \right),w_{policy} \right)$    $g\left( x\left( t \right),e_{ff}\left( t \right),K\left( t \right),y_{fb}\left( t \right) \right)\geq0$  l$\left( x\left( 0 \right),x\left( t_{f} \right) \right)=0$ | | (stochastic dynamics) (2) $\left( control policy \right) \left( SEQ Equation \backslash* ARABIC 3 \right)$  $\left( SEQ Equation \backslash* ARABIC 4 \right)$  $\left( path constraints \right) \left( SEQ Equation \backslash* ARABIC 5 \right)$  $(boundary conditions) ( SEQ Equation \backslash* ARABIC 6)$ | |

with the deterministic controls consisting of the feedforward excitations $e_{ff}\left( t \right)$and the time-varying linear feedback gain matrix $K\left( t \right), x\left( t \right)$ the stochastic states, $y_{fb}\left( t \right)$ is the stochastic feedback signal, $e\left( t \right)$ are the total muscle excitations (combination of feedforward and feedback), $w_{sys}$ is Gaussian system noise (e.g. motor noise), and $w_{policy}$ is Gaussian noise corrupting the policy (e.g. sensory noise). The system and policy noise inject uncertainty into the simulations, resulting in stochastic state variables. Consequently, all variables that depend on the state are stochastic as well. The controls, $e_{ff}\left( t \right)$ and$K\left( t \right)$, are deterministic variables.”

In the Methods section, we first formulate this general idea of system and policy noise:

“We formulate simulations of movement in the presence of noise as stochastic optimal control problems. The system dynamics are stochastic: $\dot{x}\left( t \right)=f\left( x\left( t \right),e\left( t \right),w_{sys} \right)$; with $x$ the state trajectories, $e$ the control trajectories and $w_{sys}$ a set of disturbances acting in the system with a zero-mean Gaussian distribution (noise). The control trajectories $e\left( t \right)$ are determined by the control policy:

$e\left( t \right)=e_{ff}\left( t \right)+K\left( t \right)*y_{fb}\left( t \right)$,

which is a linear time-varying feedback law parametrized by the deterministic time-varying constants $e_{ff}\left( t \right)$ and deterministic time-varying feedback gains $K\left( t \right),$which are multiplied with the feedback signal $y_{fb}\left( t \right)=h\left( x\left( t \right),w_{policy} \right). w_{policy}$ is a set of stochastic disturbances acting in the control policy with a zero-mean Gaussian distribution. Note that when $K\left( t \right)$ is non-zero, the control trajectories $u\left( t \right)$are stochastic, and depend on the state.”

Finally, we detail how these general descriptions of noise are modelled for the specific models of STANDING:

“The policy noise ($w_{policy}$) consisted of gaussian vestibular ($w_{v,q},w_{v,\dot{q}}$) and proprioceptive noise ($w_{p,q},w_{p,\dot{q}}$) with respective variance $\sigma_{v,q}^{2},\sigma_{v,\dot{q}}^{2}$ and $\sigma_{p,q}^{2},\sigma_{p,\dot{q}}^{2}$ that was added to both the vestibular and proprioceptive cues. Part of the system noise ($w_{sys}$) consisted of additive motor noise ($w_{SOL},w_{TA})$with variance ($\sigma_{SOL}^{2},\sigma_{TA}^{2}$) that corrupted the muscle activations. All sensory and motor noise sources were independent.

To simulate random rotation and translation perturbations, the system noise ($w_{sys}$) was extended. The random platform angular position and velocity were modeled as zero mean Gaussian noise $(w_{SS,q},w_{SS,\dot{q}})$ with constant variance ($\sigma_{SS,q}^{2},\sigma_{SS,\dot{q}}^{2}$).”

and REACHING:

“Policy or sensory noise was modeled by additive Gaussian noise on the end-effector position ($w_{{EE}_{x}},w_{{EE}_{y}}$) and velocity ($w_{\dot{EE}_{x}},w_{\dot{EE}_{y}}$) with respective variance ($\sigma_{EE,x}^{2},\sigma_{EE,y}^{2},\sigma_{EE,\dot{x}}^{2},\sigma_{EE,\dot{y}}^{2}$). The noisy end-effector positions and velocities were input to the feedback law. System noise consisted of motor noise that was modeled by Gaussian additive noise added to each joint torque ($w_{s},w_{e})$ with variance ($\sigma_{s}^{2},\sigma_{e}^{2}$).”

In the standing task, can the authors use some e_ff(t) (feedforward muscle excitations) rather than a_base (feedforward muscle activations) as control? Does it influence the findings about co-contraction?

To be more consistent with our general framework we changed the model to have e_ff rather than a_base. We have updated the results section + figures and methods section to reflect the changes. Note that for the simulations of standing balance, e_ff(t) results in the exact same solution as having a_base as feedforward control component, since the ‘mean trajectory’ is a static pose. Maintaining a mean static pose requires constant feedforward excitations e_ff(t), and when e_ff is constant it equals a_base.

Also, why assuming constant controls in the standing task whereas the framework allows for time-varying controls (L. 621-622)?

Although it is true that the equilibrium point for standing balance can change over long time frames [cfr. Rambling and trembling Zatsiorsky et al. 1999 in Motor Control], here we consider a single mean pose around which equilibrium is maintained. In this case, we only need to optimize a trajectory consisting of a single time point to represent a mean pose in equilibrium (state derivative is zero) and a constant covariance matrix.

We added the following phrase to the methods section - standing balance: “We thus solved for a single state, rather than a state trajectory, and the corresponding control policy to maintain a stable upright posture in the presence of noise.”

Could it be possible to introduce motor noise on muscle excitations directly (as suggested in SI) rather than on muscle forces/torques and, again, would it influence the results?

It is possible to impose noise (both motor and sensory) at any point in either the system dynamics or the control policy. We think that the adaptations in the SI and methods on the formulation of the framework make this now clear. When we consider motor noise, it could thus be imposed at the torque, force, activation or excitation level. This might influence the results as for example motor noise that is imposed at the excitation level would first pass through the delay (first order filter) before arriving at the activation level. This will have a quantitative effect rather than a qualitative effect in the sense that with different properties of the noise sources the same effects/sensitivities should arise when adding noise for example at the excitation or activation level.

Finally, as pref(t) seems to be a deterministic function of the mean trajectory in simulations (L. 750-751), does pref(t) need to be explicitly part of the optimization or could yfb be defined as yfb=h(x,xmean)? If the framework really needs pref(t), its nature and role must be better explained from the beginning.

pref(t) is indeed not a part of the optimization and we therefore removed it from all the formulations. A reference trajectory, if needed, could be part of the feedback function as suggested by the reviewer. This is in fact the case for both our simulations. For standing it is the upright position and thus there is no need to mention a reference trajectory/position and for reaching it is the (deterministic) end-effector trajectory that can be computed form the mean kinematic trajectory. This is explained in the methods section:

REACHING:

“The reference end-effector trajectory (${EE}_{ref}$) is the end-effector trajectory in the absence of noise, which can thus be computed from the mean joint trajectories

${EE}_{ref}=f_{kin}(q_{mean},\dot{q}_{mean})$”

Third, the way feedback is modeled may be somewhat confusing. This could explain the failure to predict a significant muscle co-contraction in the simulation of the divergent force field experiment (discussed L. 467-489). The reason for this failure thus deserves to be clarified because previous simulations assuming feedforward-only control had predicted muscle co-contraction for unstable tasks with a similar framework (1).

The reason we predicted less co-contraction than previous work is that we simultaneously solved for feedforward and feedback control. In a feedforward-only context, muscle co-contraction is the only means of stabilizing the system and meeting the task constraints in terms of accuracy (either as part of the cost or as constraint). If there is both feedforward and feedback, the contributions of feedforward and feedback will depend on their relative cost (in our case in terms of expected effort). We have following paragraph in the discussion:

“Muscle co-contraction has been observed experimentally during reaching [75], especially in a divergent force field [63], [65], [66], but was not predicted by our stochastic optimal control simulations. Berret et al. found higher levels of co-contraction during reaching, in the absence of feedback control [35]. Co-contraction will increase joint impedance and hence robustness against perturbations during reaching. Yet our simulations suggest that this is an energetically costly strategy when feedback is present. In addition, there is experimental evidence for both co-contraction and feedback corrections during reaching.”

Admittedly, several modeling assumptions might have resulted in unrealistically efficient feedback and thus an underestimation of co-contraction. First, we use a first order approximation of the delays instead of real delays (see next comment). Second, in the reaching simulations feedback gains could change instantaneously as a function of time, whereas this infinite bandwidth is probably not realistic. We added to the discussion:

“Third, feedback gains were unconstrained in how they could change as a function of time. Such infinite bandwidth is probably unrealistic and limiting this would likely result in less efficient feedback control and increased levels of co-contraction.”

Here the current lack of co-contraction might be related to an overestimated efficiency of high-level feedback control in this task because state estimation processes and sensory delays are not explicitly modeled. Although the authors explain that the time constant (tau) of the first-order muscle dynamics “lumps together sensory and motor delays”, it is still different from a feedback controller using delayed sensory measurements. Here, yfb(t) is known instantaneously from the current state x(t) and it thus affects muscle excitations (control) instantaneously. The constant tau mainly specifies that muscle excitations cannot modify muscle force instantaneously but does not specify that the controller has no access to the current state (and must infer it). Thus, in a divergent force field, the brain may not know the current hand’s state as accurately as assumed by the present modeling, which could explain why muscle co-contraction is observed in experimental data. Therefore, would the model predict more muscle co-contraction if one assumes more uncertainty about yfb(t) in the divergent force field task or considers explicitly a delayed feedback signal yfb(t-delay)?

We agree that the lack of predicting muscle co-contraction might be due to having a too efficient feedback control scheme. Here, efficiency might be overestimated by having no exact delays as well as by allowing feedback gains to change instantaneously in time. We think that we can address both shortcomings with our framework but considered it out of the scope of this paper. For example, we could constrain how fast feedback gains can change in time.

We have a paragraph considering the modelling assumptions (including how we model feedback) that could affect predictions of muscle co-contraction:

“Several modeling assumptions might have influenced the simulated levels of co-contraction. First, we did not model that co-contraction increases short-latency reflexes through gain scaling [76], [77], thereby underestimating the contribution of co-contraction to reflexes, potentially underestimating co-contraction. Second, due to the first order approximation to model feedback delays, sensory information – although attenuated - is available immediately. This might lead to underestimating co-contraction as the efficiency of feedback corrections is increased. Note, however, that the presence of sensorimotor noise prevented simulated feedback gains and therefore ‘early’ feedback contributions from becoming too large. Third, feedback gains were unconstrained in how they could change as a function of time. Such infinite bandwidth is probably unrealistic and limiting this would likely result in less efficient feedback control and increased levels of co-contraction. Fourth, our framework does not allow predictions of the agonist-antagonist dual control strategy which has been suggested as an important reason to perform co-contraction, thereby potentially underestimating co-contraction. By co-contracting antagonistic muscles, it is possible to increase agonist activity and decrease antagonist activity simultaneously yielding a more efficient response to a perturbation. However, our framework does not allow predictions of this agonist-antagonist dual control strategy depending on muscle co-contraction [60], which might explain why we underestimated muscle co-contraction. Although our approach to stochastic optimal control accounts for nonlinear state dynamics, it was still based on a linear approximation around the mean state trajectory to propagate the state covariance matrix. An important nonlinearity arises from muscle activity being bound between zero and one and hence, reductions in muscle activity are only possible when muscle activity is larger than zero. Yet this nonlinearity is not reflected in our approximation of the state covariance dynamics, which results in predicted reductions in muscle excitations if warranted for a perturbation, even when this would result in muscle excitations becoming smaller than zero. Hence, there is no need to increase baseline activity in our simulations to exploit the agonist-antagonist dual control strategy, which likely resulted in overly large predicted contributions of this strategy as they came at no cost. Finally, we used an all or nothing approach to model short-range-stiffness whereas short-range-stiffness disappears when muscle stretch exceeds a certain threshold [48]. Therefore, short-range-stiffness might only contribute during low amplitude sway up to 3° [40], [48], and we might have overestimated its contribution when platform perturbations were high enough to induce large sway. Similarly, we might have overestimated contributions from co-contraction by modeling rigid tendon, especially when accounting for short-range stiffness (see also Supplementary information). Although the level of co-contraction might be sensitive to each of the above assumptions, our simulations that simultaneously solved for optimal feedforward and feedback control contributions demonstrated that both muscle co-contraction and feedback gains varied as a function of the task, mechanical properties of the muscles, and sensory acuity. ”

By extension, it is unclear whether the modeled feedback indirectly captures the effects of long-latency or short-latency reflexes, or a mix of both. The fact that the feedback signal yfb involves task-level variables (e.g., end-effector position/velocity) points toward a supraspinal processing (thus long-latency reflexes). However, would the results change if feedback signals involved only joint positions/velocities (i.e., q and qdot directly, without the forward kinematics mapping)? If they don’t, together with the absence of explicit modeling of sensory delays and state estimation process, this could rather point toward short-latency spinal reflexes, the gains of which increase with muscle co-contraction as noted by the authors (L. 487-489). If these considerations can affect the interpretation of K(t) and its contribution to impedance control in the proposed model, this should be better tested and stressed throughout the paper.

The behaviors that our simulations reproduce can only be explained by long-latency responses in both standing balance control and in reaching perturbations. Specifically, maintaining upright balance to support-surface rotation perturbation cannot be explained by short-latency responses that maintain the posture of the joints (Carpenter et al. 1999 in Experimental Brain Research). Instead, the long-latency balance correcting response activates the opposite set of muscles as those recruited by the short-latency response that work to maintain body posture. This is shown in Figure 1 where the vestibular loss simulations fail to stabilize the body at larger perturbations when only using proprioceptive signals for balance (Peterka 2002 in Journal of Neurophysiology). Similarly, during reaching, the complex changes in perturbation responses based on task-level goals have been shown to be a long-latency response phenomenon (Cluff et al. 2015 in Vision Research) requiring changes in multi-joint control.

We realize we do not have the most accurate sensorimotor map especially because of simplification in the sensory dynamics. The purpose of this example is rather to show that principles of motor control that have been suggested by simulations with ‘simple’ models still hold and that the inclusion of more accurate models increases the fidelity of the predictions. Our model can surely be improved (e.g. separating long- and short-latency reflexes). This is future work, in which hopefully other researchers will participate while relying on the presented computational framework.

We added a short sentence in the beginning of the results to clarify that we are modelling task-level feedback rather than separating short and long latency reflexes:

“We did not separately model short- and long-latency reflexes and considered task-level sensory feedback rather than local sensory feedback.”

Hand velocity profiles should be depicted to fully visualize trajectories in the reaching tasks. Also, it could be interesting to plot the optimal deterministic controls (in a supplementary file if needed).

Thank you for this suggestion. We added a plot in the supplementary information (figure S1) that contains the mean trajectories with confidence ellipses for variability, velocity profiles and optimal deterministic controls.

Could you please detail the derivation of Eq. 28 from Eq. 1 in the SI? The reason why cross terms disappear was not clear to me.

Thank you, we have now updated the SI with the following :

Equation (28) can be derived from equation (1) since deterministic variables are independent from stochastic variables ($E\left[ e_{ff}\cdot e_{fb} \right]=E[e_{ff}]\cdot E[e_{fb}]$), the expected value of the feedback signal is zero ($E\left[ e_{fb} \right]=0$), all noise sources are independent, and the variance of deterministic variables, such as the feedforward muscle excitations $e_{ff}$, is zero ($Var\left( e_{ff} \right)=0$):

$$E\left[ e^{2} \right]=E\left[ e_{ff}^{2} \right]+E\left[ e_{fb}^{2} \right]+2\cdot E[e_{ff}\cdot e_{fb}]$$

$$E\left[ e^{2} \right]=E\left[ e_{ff} \right]^{2}+Var\left( e_{ff} \right)+E\left[ e_{fb} \right]^{2}+Var(e_{fb})+2\cdot E[e_{ff}]\cdot E[e_{fb}]$$

$$E\left[ e^{2} \right]=E\left[ e_{ff} \right]^{2}+0+0+Var(e_{fb})$$

$$E\left[ e^{2} \right]=E\left[ e_{ff} \right]^{2}+Var\left( K\cdot y_{fb} \right)+Var\left( K\cdot w_{s} \right)+Covar(K\cdot y_{fb},K\cdot w_{s})$$

$$E\left[ e^{2} \right]=E\left[ e_{ff} \right]^{2}+tr(K\cdot Var\left( y_{fb} \right)\cdot K^{'})+tr(K\cdot\Sigma_{w_{s}}\cdot K^{'})+0$$

where $Covar\left( K\cdot y_{fb},K\cdot w_{s} \right)=0$ as the noise sources are independent.

*.*

Also, is Eq. 38 in SI based on a Taylor expansion?

Yes, eq. 38 is based on a linearization as well, we added it to make this clear.

Note further that through linearization we can write:

$$Var\left( y_{fb,k} \right)=\frac{\partial h\left( x_{mean,k}, \right)}{\partial x}P_{k}{\frac{\partial h\left( x_{mean,k}, \right)}{\partial x}}^{T}$$

(38)

Note that the SI file still contains some comments from the authors. I suggest to carefully review all equations within. I did not check all equations there because they are more secondary to the article, but I am not sure that M_k is defined in Eq. 30 and the following.

Thank you for pointing out these comments. We have made a full review of the equations

Mk is a helper variable that could be removed from the formulation. However it is beneficial for numerical efficiency to add this term (avoiding inversion of another matrix expression that might be ill-conditioned). We added this to the SI:

… “With $M_{k}$ a helper variable introduced to avoid the inversion of the matrix ${\left( \frac{\partial G}{\partial z} \right)_{k}}^{T}$”

Throughout the paper, the modeling work involves stochastic differential equations but uses the formalism of ordinary differential equations. Why?

- We used a similar notation as in the thesis of Gillis et al. and the conference paper of Houska et. al on which we built our framework.
- Also, because papers in our field are typically described by ODEs, this seemed easier to demonstrate how our approach extends current computational methods to simulate movement.

Figures 2 and 3 seem to have been inverted. Please check the captions as well. In Fig 2B, please indicate the experimental conditions for each graph.

Thank you, we have corrected this error.

L. 527: the dynamics was denoted f(x,e,w) in the Results and becomes f(x,u,w) here. Why not introducing u(t) already from the Results?

We now only used e(t)

L. 537: Why is there a prime in Eq. 2 for sigma_w? If it is the transposition operator, please note that it is also denoted by T elsewhere.

- In accordance with notations used by Gillis et al. and Houska et al., we used the prime to denote that this is the continuous time representation of the noise sources.
- We added following sentence following eq.2: “with Σ_w' specifying the continuous time (denoted by ') covariance matrix describing the noise sources.”
- We have checked that the transpose operator T is used where appropriate.

In Eq. 5 (L. 541), the expectation is not needed if all the quantities are deterministic. Please note that J is already used L. 166 to denote the integral cost (not integrand).

- Thanks, we removed the expected cost and replaced J with another symbol to denote another function.

Please indicate the time interval in the integrals (e.g. L. 166), and mention if the optimal control problems are considered in finite horizon or if the framework is also considered in infinite horizon (as asymptotic stability is mentioned in the standing task?).

We have now indicated the time interval in the integral. The problems are always solved for a finite horizon. Standing is a special case as mentioned earlier. We simulated standing using a single time point as the mean pose was static (state derivative is zero) and the covariance matrix was imposed to be constant to ensure marginal stability. The reach simulations are finite horizon with a selected simulated time of 800ms.

We were in fact not fully correct in using asymptotic stability. We actually impose marginal (periodic) stability for the given noise sources. We have edited the manuscript accordingly.

To clarify the formulation of the standing balance problem we changed the paragraph in the Methods section:

“The task-goal during standing was to maintain a stable upright posture (marginal stability). Postural marginal stability, in the presence of noise, was modeled by constraining the mean angle of the pendulum to be upright with zero angular velocity, the mean state derivatives to be zero (mean pendulum position and velocity and muscle activations are constant in time), and the state covariance derivatives to be zero: $\dot{P}=0$ (state covariance matrix was constant in time). We thus solved for a single state, rather than a state trajectory, and the corresponding control policy to maintain a stable upright posture in the presence of noise.”

L. 403-405: this statement is not true if we consider the references below (1-3)

We believe the reviewer is referring to the following sentence: “Most prior methods have only allowed efficient solutions of linear, but not nonlinear, stochastic optimal control problems [6]–[8], [61], so that they were not capable of predicting many important variables causing movement”

We have now changed the paragraph to incorporate the relevant references and better point out the differences between the presented work and previous work: “Implementing a direct collocation approach to the solution of nonlinear stochastic optimal control problems increased computational efficiency and modelling flexibility, allowing neuro-musculoskeletal model complexity, and thereby fidelity, to be increased. Most prior methods have only allowed efficient solutions of linear, but not nonlinear, stochastic optimal control problems [7]–[9], [69], and were therefore not capable of predicting many important movement features. Stochastic optimal control simulations based on nonlinear dynamics have previously been performed using iterative Linear-Quadratic-Gaussian (iLQG) methods for reaching tasks [19]. However, such methods require a time-marching integration of the system dynamics under the current guess of the control law at each iteration to evaluate the nominal controls and state trajectories. Such shooting methods might be less suitable than collocation methods for movement tasks with less stable system dynamics, such as standing or walking. Further, the LQG approach assumes a quadratic cost function and a linear and continuously time-varying feedback control law, which might not always represent the biological system. Finally, LQG methods require constraints on the mean state and state covariance (e.g. limiting the standard deviation of the end-point position of the hand in a reaching task) to be imposed through penalty terms in the cost function, whose weights require tedious tuning. In contrast, the approach based on collocation and gradient-based optimization we used here allows imposing such constraints in a direct and intuitive manner. A few previous studies [70], [71] have relied on direct collocation methods as well to solve stochastic optimal control problems but they evaluated the stochastic cost function and path constraints based on the simulation of a limited number of noisy episodes rather than describing the state distribution. For nonlinear systems with a limited number of degrees of freedom, such a sampling based approach appears tractable. However, with increasing degrees of freedom, the number of noisy episodes that is required to capture the underlying stochastic dynamics might become computationally intractable. Simulations based on too few episodes might result in problematic unstable and sub-optimal solutions. We overcame the limitations of prior methods by applying a recently proposed method to approximate stochastic optimal control problems by deterministic optimal control problems [30], [31]. The proposed approach is based on the assumption that the state distribution can be approximated by a Gaussian distribution and that the propagation of the state covariance can be described by the Lyapunov equation [32][32]. This framework is applicable to optimal control problem formulations with any cost function design while feedback laws with any temporal and structural design are possible. Here, we used it to simultaneously solve for feedforward and feedback control laws. In addition, the possibility to impose path constraints on both the mean state and state covariance trajectories allows for an intuitive formulation of task requirements such as the desired accuracy.”

L. 490-492: but see previous works (1-3)

We acknowledge the referred papers, but state the difference with what we did here in the following way: “A major contribution of our work was to create a framework for predicting how stochastic optimal control principles apply at the level of individual joints and muscles, which has only recently been done in one other study [32] that did not include feedback control.”

L. 501-504: this was already observed in (1)

Thanks for pointing this out. We know mention this in the paper and discuss how our results our different. In (1) only mechanical stiffness resulting from the intrinsic muscle properties was assessed whereas measured stiffness typically contains contributions from mechanical stiffness and neural control as the displacement is measured after 50ms to 100ms following a perturbation. We assessed stiffness similar as in experiments. Therefore, our stiffness ellipses capture the effect of feedforward (activation dependent muscle stiffness) and feedback control.

We added following sentence to the discussion: “Modulation of stiffness ellipses in force fields have been shown previously for a non-linear muscle driven arm model [32]. However this study neglected contributions of neural feedback and computed stiffness ellipses differently from how these are measured experimentally.”

L. 592-596: I did not understand this paragraph well. Is it to specify the constraints of the optimal control problem? Why using these assumptions that seem to be quite strong? Please clarify.

It is indeed to specify constraints that guarantee a system with controller that induces marginal stability given the injected noise. I suppose the reviewer means that imposing $\dot{P}=0$ is restrictive. But, note that the mean state (x) and the state covariance matrix (P) are optimization variables as well. In that sense, I think the assumptions are not so restrictive, it are the minimal constraints that make sure the simulation won’t fall and is in a stable regime.

A solution that would result in $\dot{P}\prec0$ would also guarantee stability and is less restrictive. However in that case there is ‘smaller’ covariance matrix P for the same feedback law that would stay constant in time, resulting in a lower cost function.

L. 689: why not considering biarticular muscles since the model and methods allowed it? Then, the authors could have directly reused existing neuromechanical models without having to adjust muscle parameters. I did not get the reason why such a simplification was implemented here.

Based on your and other reviewers’ comments we repeated the simulations with an arm model driven by 6 muscles (Todorov 2003). This model differed substantially from our previous model in that the muscles had different moment arms and lengths. Also we now used the traditional (unadapted) muscle force-length characteristics as described in De Groote et al. (2016). In the previous model, these were adapted to make sure the muscles, that were a subset from the muscles in a more complex model, would operate on an acceptable region of the force-length curve.

In the main text we now only report the results with the new 6-muscle model.

The reach trajectories differ from the previous model (especially the perturbed trajectories for the circle and bar condition). In summary, the perturbed reach trajectories for the circle condition became more realistic as the overshoot that was not observed experimentally disappeared whereas the perturbed reach trajectories for the bar condition became less realistic as the overshoot that was observed experimentally also disappeared. For completeness, we added a comparison in the SI to show how the changes in the muscle model affect the predictions. We compared three models:

1. 6 muscle model (Todorov 2003,2005)
2. 4 muscle model (Todorov 2003,2005 but with biarticular muscles removed, other muscles unchanged)
3. 4 muscle model as described in the previous submission

Our reach trajectories with 6 muscles were not substantially different from simulations with 4 muscles (models 1 and 2).

Hence, differences in muscle parameters and not the addition of biarticular muscles explain the differences in reaching trajectories.

From these simulations, it is clear that muscle parameters play an important role.

We added the following to the discussion to refer to these additional simulations:

“While it is known that using different levels of detail to represent the musculoskeletal dynamics lead to different simulated responses to perturbations if motor commands remained unchanged [78], optimal control predictions inform how the nervous system can exploit the nonlinear dynamics of the musculoskeletal system. As such the ability of stochastic optimal control to predict movement cannot be accurately validated using simple models. For example, our two-segment, muscle-driven arm model yielded more realistic reach trajectories when reaching to different targets, when compared to prior predictions based on a point mass [9].  We further explored the sensitivity of predicted reach trajectories to the presence of bi-articular muscles and muscle properties (see Supplement for details). Interestingly, simulated reach trajectories were robust against the removal of bi-articular muscles but sensitive to how mono-articular muscles were modeled. Using an alternative, physiologically plausible set of muscle properties yielded perturbed reach trajectories in the ‘bar’ condition that captured the experimentally observed overshoot. However, the overshoot was also predicted for perturbed reach trajectories in the ‘circle’ condition, where it was not observed experimentally. These exploratory results demonstrate both the potential of our framework to explore the effect of musculoskeletal properties on (perturbed) movement and the importance of accurate musculoskeletal models.”

Please find our simulation results comparing these 3 models in the supplementary information

L. 745: "Tmotor the stochastic torque acting at shoulder and elbow resulting from motor noise ws,we.". This sentence sounds strange as motor noise is added to Tmotor. Please verify.

- Thanks for pointing this out, we removed Tmotor

**REVIEWER 2**

1. Lines 45, 46. Suggest ‘. . . to be identified since task constraints . . . penalty terms in a cost function.’

- Thanks you, we have implemented the suggestions.

2. Line 64, Suggest ‘. . . to aid . . .’

- Indeed, this word was missing

3. Lines 105. 106. There are many mentions of ‘collocation’ and ‘shooting methods’ throughout the manuscript beginning on these lines. While the given reference [5] provides a window into learning more about what exactly are these methods, this paper would be more understandable if some definition or description of what is meant by these terms are provided. Perhaps an appropriate place would be in the Supplementary material or possibly the Discussion.

- We do feel that shooting and collocation methods are well established and that interested readers will therefore find ample references and tutorials (including the provided references). We therefore refrained from giving a detailed description of these methods in the supplement. However, to help the readability of the paper, we extended the introduction . In addition, we provided additional references.
- “The introduction of direct collocation approaches to solve trajectory optimization problems improved computational efficiency compared to shooting methods by decreasing the sensitivity of the optimization objective to the decision variables [22]. Shooting methods use time-marching integration to evaluate the cost and constraint function based on the current guess of the initial state and controls. Due to time-marching integration over the entire movement horizon, shooting methods have poor convergence and long computation times when the dynamics are stiff, as in many biological movements. In contrast to direct shooting, direct collocation eliminates the need for time-marching integration by adding the parameterized states to the decision variables and by adding the discretized dynamic equations as constraints to the optimization [23].”

4. Line 127. References [27-[29] are given for Houska. Is this correct? Or should it be [25]-[27]?

There was indeed a mistake. We corrected the references..

5. Line 194. This is the first mention of short-range stiffness. The acronym SRS should be defined here.

done

6. Line 200. Should be ‘. . . noise was added to . . .’

done

7. Line 222. Reference [47] is a reference to a very general neuroscience textbook. Please use a more appropriate reference like [31]

Reference was added

8. Line 229. CCI was not previously defined and is only defined in the Figure 1 legend. It should be defined here. Similarly, SRS was not defined previously. Also, the labels ‘healthy-SRS’ and ‘VL-SRS’ used here and in Figure 1 is somewhat confusing since the ‘-‘ could be interpreted as a minus sign in the sense that the model is subtracting out the SRS component rather than including it. Suggest maybe ‘healthy with SRS’ and ‘VL with SRS’ be used here and in Figure 1. Also, this labeling is used in the Methods section lines 609, 610.

we added the necessary labeling and changed to ‘… with SRS’ to improve clarity

9. Lines 243, 244. This is an interesting prediction regarding a shift toward increased use of vestibular information for balance with increasing platform translation amplitude. This is an example of model prediction that could potentially be tested in future experiments to test the validity of the prediction (and the methods used to make the prediction) as well as revealing something new about balance control. You might want to highlight this in the Discussion.

Thanks, we added a sentence in the results immediately, as this is easier for the reader than to come back in the discussion to such a specific prediction: “Such decreased contribution of proprioceptive information with increasing translation magnitude has not been tested experimentally and might be interesting to validate the optimal control assumption and underlying model.”

10. Figure 1. For the experimental rotation results showing in part A, there are experiment sensory weight measures (the Kproprio/( Kproprio+Kvest) measure) from four VL subjects as a function of rotation magnitude (Figure 10 in reference [31]). These could be shown and would be in agreement with the model-predicted results.

We do not think the figure from the cited represents what we show here. Figure 10 of [31] shows the trade-off between the visual and somatosensory system whereas we did not model the visual system (cfr. blindfolding subjects). In our simulated experiment: blindfolded platform rotations/translations with VL subjects, there is no sensory reweighting possible from VL subjects. The experimental curve from the healthy subjects is actually based on chapter 41: Posture in the book by Kandel et al. (Principles of Neural Science). Here they report to have an adapted figure from reference [31].

11. Figures 2 and 3. I note that in the reviewer download of the manuscript pdf Figures 2 and 3 were reversed, but not in the pre-print pdf.

Thanks for noting this. We have corrected this.

12. Figure 2. In part B the ‘Reaching accuracy’ plots should be centered below their respective reaching trajectories.

Done.

13. Figure 3C. The vertical line indicating the perturbation time for the Posterior Deltoid is missing.

- We added the line.

14. Line 385. Should be ‘. . . to trade them off . . .’

- We made the edit.

15. Equation 8. In comparison to equation 10 in the Supplementary Information, it’s not clear why the lambda variable in equation 8 has an ‘i’ subscript. The information given at the end of the following paragraph suggest that lambda would have a fixed value.

- this parameter can have a different value depending on the constraint. We changed it in the SI to have a subscript ‘i’ as well.
- A simulation might require stricter ‘bounds’/offsets on different constraints.

16. Line 648. Should be ‘Table 2’.

- yes, thanks

17. Line 656. No et al. in reference [31].

- done

18. Line 671 Table description. It’s unusual to have such a long table description. Suggest that this description would be better made a part of the text since it also applies to the reaching simulations (or any system that is simulated).

- agreed. We made it part of the text.

19. Line 580 and 696. Is this ‘first-order dynamics’ with 150ms time constant meant to represent or approximate actual time delays in a closed loop feedback system because it’s not possible to represent a true time delay in their model formulation? If so, it would be informative for the authors to state whether their methods allow for the inclusion of a true time delay and thus necessitating use of some approximation.

- Although we used a first-order delay, our method can be extended to include an exact delay. This is important future work, as we now indicate in the discussion.
- We added to the discussion: “Second, due to the first order approximation to model feedback delays, sensory information – although attenuated - is available immediately. This might lead to underestimating co-contraction as the efficiency of feedback corrections is increased. Note, however, that the presence of sensorimotor noise prevented simulated feedback gains and therefore ‘early’ feedback contributions from becoming too large. Including an exact delay within the framework is important future work, and approximating delays by a low-pass filter must be done with caution when drawing conclusions.”

20. Lines 731, 732. This is text left over from the rotation section and should be deleted.

- oh yes. thanks for catching this error

21. Reference section. There are numerous references with incomplete information including 2, 3, 18, 25, 27, 28, 54, 56, 63, and 65.

- we thoroughly checked our references to make sure they are complete in the revised version.

**REVIEWER 3**

This manuscript presents a novel method to perform predictive movement simulations using neuromusculoskeletal system models that include random perturbations, motor noise, and sensory noise. The method is applied to standing, where the effect of perturbations, muscle properties, and sensory loss on the optimal neural control parameters is predicted. The method is also applied to reaching movements, to predict the optimal trajectories and the corresponding feedback-feedforward control strategies.

This is a very exciting manuscript that addresses an important problem. Experiments have suggested that movement trajectories and muscle control are affected by noise and perturbations. Traditional optimal feedback control theories for linear systems are unable to explain and predict this. There has not been an effective method for systems with nonlinearities such as occur in muscle mechanics. This paper could have a large impact on the field and encourage new applications of this approach.  Unlike previous work, this new approach has the potential to be scalable to larger musculoskeletal systems and more complex tasks.

However, my enthusiasm is diminished because the authors try to do too much, which has led to some significant weaknesses, errors, and unnecessary clutter. This may leave the reader unconvinced and lost. It is hard to see the great fundamental contribution when distracted by these weaknesses.

I would love to see a thorough rewrite of the paper, and perhaps focus on the essentials, without trying to simulate vestibular loss or short-range muscle stiffness, or too many separate noise contributions. The main goal should be to present the method and convince the reader that it works, and that the predictions make sense and agree with experiments.

Thank you for the feedback. We have worked to clarify the key contributions of the methods developed and their general applicability a variety of motor control problems. As the reviewer comments, the work is scalable to more complex musculoskeletal systems and is generalizable to different problems. Based on enthusiasm from other reviewers and as a counterpoint to prior studies using optimal control formulations that only work or are tested in a specific context, we find it more convincing that the same framework can work for a variety of questions about both the neural and musculoskeletal systems and how thay shape movement control.

Nevertheless, we agree that the initial manuscript was a bit confusing and failed to highlight the main ideas common across all of the simulations. We have thoroughly rewritten many sections of the manuscript to make the innovations of the framework more clear. We hope to engage readers from different areas of motor control so they can consider familiar examples and be convinced of the generalized utility of the approach.

A major change with respect to the previous submission is that we changed the musculoskeletal model for the reaching task to include 6 muscles (4 uniarticular and 2 biarticular). We have also moved several analyses to the supplemental information.

Specific comments follow below.

1.      I will start with the main weakness which almost made me ignore everything else. The cost function (9) is constructed in a manner that favors an equal distribution of effort between feedforward and feedback. For example, if a static posture requires a constant 50% activation level in just one muscle, it could be generated with feedforward or feedback, or any combination of both. The only difference would be the stability of the system which only plays a role in the stochastic case. However, with this cost function, the cost would be 0.25 (0.5^2) when using only feedback, and 0.25 when only using feedforward, but only 0.125 (0.25^2 + 0.25^2) when using equal amounts of feedforward and feedback. Hence that solution would be optimal, and this has nothing to do with stochastic dynamics. The cost function should clearly be related to total activation squared, not separated into feedback and feedforward components. The work should be repeated with the proper cost function to avoid favoring certain solutions.

The cost function can be decomposed in the separate contributions between feedforward and feedback muscle activities (or excitations) and starts from total activation squared.

We added a derivation in the SI: “Equation (28) can be derived from equation (1) since deterministic variables are independent from stochastic variables ($E\left[ e_{ff}\cdot e_{fb} \right]=E[e_{ff}]\cdot E[e_{fb}]$), the expected value of the feedback signal is zero ($E\left[ e_{fb} \right]=0$), all noise sources are independent, and the variance of deterministic variables, such as the feedforward muscle excitations $e_{ff}$, is zero ($Var\left( e_{ff} \right)=0$):

$$E\left[ e^{2} \right]=E\left[ e_{ff}^{2} \right]+E\left[ e_{fb}^{2} \right]+2\cdot E[e_{ff}\cdot e_{fb}]$$

$$E\left[ e^{2} \right]=E\left[ e_{ff} \right]^{2}+Var\left( e_{ff} \right)+E\left[ e_{fb} \right]^{2}+Var(e_{fb})+2\cdot E[e_{ff}]\cdot E[e_{fb}]$$

$$E\left[ e^{2} \right]=E\left[ e_{ff} \right]^{2}+0+0+Var(e_{fb})$$

$$E\left[ e^{2} \right]=E\left[ e_{ff} \right]^{2}+Var\left( K\cdot y_{fb} \right)+Var\left( K\cdot w_{s} \right)+Covar(K\cdot y_{fb},K\cdot w_{s})$$

$$E\left[ e^{2} \right]=E\left[ e_{ff} \right]^{2}+tr(K\cdot Var\left( y_{fb} \right)\cdot K^{'})+tr(K\cdot\Sigma_{w_{s}}\cdot K^{'})+0$$

where $Covar\left( K\cdot y_{fb},K\cdot w_{s} \right)=0$ as the noise sources are independent.”

2.      The Introduction may benefit from reminding readers that linearization will produce the same optimal control law, and the same optimal trajectory, regardless of the amount of noise in the system (due to the Certainty Equivalence principle). Linearization would never predict that trajectories and control laws depend on noise level, as suggested by experiments and by the authors’ novel optimization method. A good example of linearization is: He, Levine & Loeb, IEEE Trans Autom Control 1991.

Thank you for this comment. We believe that the reviewer’s assertion is based on a limited set of problem formulations. In our framework, we implemented the task goal as a constraint rather than as a quadratic cost. In this case it is possible for noise to alter the control law. In addition, changing the relative level of noise in different sensory signals that are combined in the feedback law (i.e. sensory reweighting), will also affect the optimal controller.

3.      Line 198, “delayed proprioceptive and vestibular cues”. As far as I can tell, the feedback control (eq. 14 in Methods) does not have time delay.

Thank you for pointing our this error. We have made this more precise and clearly state that we lack an exact time delay in the Discussion. We kept the word “delayed” but clearly state this is a first order delay mapping the excitation to activation signal.

We now clarify implications of our approximations for delays in the discussion:

“Second, due to the first order approximation to model feedback delays, sensory information – although attenuated - is available immediately. This might lead to underestimating co-contraction as the efficiency of feedback corrections is increased. Note, however, that the presence of sensorimotor noise prevented simulated feedback gains and therefore ‘early’ feedback contributions from becoming too large. Including an exact delay within the framework is important future work, and approximating delays by a low-pass filter must be done with caution when drawing conclusions.”

4.      Line 228, “moderate levels”.  If I read the graph correctly, the CCI is 2% which seems low, rather than moderate.

We agree that “low” is a more appropriate description than “moderate” co-contraction and have edited this point as follows:

“During platform rotations, slightly higher, but still low levels of co-contraction were predicted for vestibular loss subjects [57] than for healthy controls …”

5.      Line 259. I feel that the work would be much stronger if a more conventional Hill model was used, with a series elasticity rather than a rigid tendon, and without the added short range stiffness. A nonlinear series elasticity would create a force-dependent stiffness, and it would be truly a short range stiffness because the stiffness decreases as soon as the contractile element “catches up” with an external stretch (e.g. GK Cole et al., J Biomech 1996).  Series elasticity can be seen as the combined elastic properties of tendon, aponeurosis, and cross-bridges. The SRS model may give the impression of an artificial “trick” to introduce a benefit of co-contraction. As modeled in eq. (10), “short range” is a misnomer. The stiffness is only dependent on activation and not on the amount of stretch. It would be best to eliminate this controversy and show that predictions can be obtained with a conventional Hill model. Otherwise, readers may think the method is not applicable to the usual musculoskeletal models.

Although including a tendon could indeed cause a nonlinear stiffness, it has a very different characteristic and is of much smaller magnitude to the short-range stiffness observed in muscle fibers. We have now added a discussion in the SI to show how adding a would change our simulation results. Including a compliant tendon would likely affect results only if we use the augmented Hill-type model that includes the activation dependent stiffness model that we use to account for the short-range stiffness properties of muscle.

We use “short-range stiffness” to refer to the muscle phenomenon and not how it is modeled. Although our phenomenolocial model is not dependent on muscle stretch, the biophysical process arises from detaching crossbridges when the muscle is stretched. During postural perturbations, this process has a fairly stereotyped behavior that we model based the trajectory of the stiffness derived from that seen in single, permeabilized, muscle fibers in calcium solution, where noncontractile tissues have been removed (Getz et al. in Biophysics Journal 1998). We used this previously to demonstrate the necessity of augmenting Hill-type models with muscle short-range stiffness to appropriately reproduce the musculoskeletal system’s response to postural perturbations (De Groote et al 2017). simulations of postural control. While this very simplified may appear to be a “trick”, but it is a reasonable representation of the muscle’s short-range stiffness property and is indeed necessary to obtain the benefits of muscle co-contraction. It is well-known that muscle response to stretch has a much steeper F-L curve than the steady-state F-L curve represented in the Hill model (Rack et al. 1974, Shue and Crago Annals of Biomedical Engineering 1998 ). As such, any Hill-type model will require very high co-activation on the rising part of the F-L curve (Bunderson et al 2008) and unlikely to predict muscle coactivation. While we currently do not have the stretch-dependent property of short-range stiffness, we demonstrate how a transient, activation dependent stiffness might be exploited under specific circumstances to minimize effort.

Current model formulations for short-range stiffness are based on biophysical mechanisms and are not yet in a form that can be used in optimal control formulations. However, in our ongoing work, we aim to develop more realistic models of muscle response to stretch.

We refer to the rigid tendon assumption in the discussion the following point to our discussion:

“Similarly, we might have overestimated contributions from co-contraction by modeling rigid tendon, especially when accounting for short-range stiffness (see also Supplementary information).”

Section in the SI on the rigid tendon assumption:

In this section we aim to provide more insight in how the rigid tendon assumption might have affected the results. Note that using rigid tendons resulted in algebraic contraction dynamics and simplified the optimal control problems. However, the framework is sufficiently general to consider compliant tendons in future work. Adding a compliant tendon in series to the muscle fibers will reduce the compliance of the muscle-tendon unit. This will have a large influence on muscle-tendon stiffness when the muscle is stiffer than the tendon as in our simulations of standing balance with short-range stiffness, but a small influence on muscle-tendon stiffness when the muscle is more compliant than the tendon as in our simulations of standing balance and reaching with a Hill-type muscle model. Here, we illustrate this for the soleus muscle. We chose the soleus, as it has a longer and thus more compliant tendon than the other muscles we modeled. Therefore, the effect of adding a compliant tendon is expected to be largest for this muscle.

We computed stiffness of the muscle fibers as $k_{muscle}=\frac{{\partial F}_{m}}{\partial l_{m}}cos(\alpha)$ with $F_{m}$ the muscle fiber force, $l_{m}$ the muscle fiber length and $\alpha$ the pennation angle; stiffness of the tendon as $k_{tendon}=\frac{{\partial F}_{T}}{\partial l_{T}}$ with $F_{T}$ the tendon force, $l_{T}$ the tendon length. Stiffness of the muscle-tendon unit was computed as: $k_{MT}=\left( \frac{1}{k_{tendon}}+\frac{1}{k_{muscle}} \right)^{-1}$. Note that when assuming a rigid tendon $k_{MT}=k_{muscle}$.

We first discuss the observations for a soleus Hill-type muscle without short-range-stiffness. In the rigid tendon case, the length of both the fiber and tendon is the same for every activation level (Figure 3 A – length, dotted lines). In the rigid tendon case, $k_{muscle}$, and thus $k_{MT},$ increases linearly with muscle activation level as the muscle is operating on the ascending limb of the force-length relationship (Figure 3 A - stiffness, green dotted line). In the compliant tendon case, the fiber length decreases for increasing activation, changing the operating point of the muscle on the force-length relation, and the tendon length increases for increasing activation (Figure 3 A – length, solid lines). As a consequence, $k_{muscle}$ (Figure 3 A – stiffness, blue solid line) increases at a superlinear rate with activation level as muscle fiber length decreases with increasing activation level shifting the muscle’s operating length to a steeper part of the force-length relation in this specific case. However, differences in muscle stiffness when considering a compliant instead of a rigid tendon are small (Figure 3 A – detail MT stiffness). The compliant tendon (Figure 3 A – stiffness, red solid line) is much stiffer than the muscle fibers (at least factor 6), and as a result has a very minor effect on $k_{MT}$ (green solid line). In Figure 3 A – detail MT stiffness, we see that at high muscle activations (above 0.75), muscle-tendon stiffness becomes higher for the compliant than for the rigid tendon, because the increase in fiber stiffness due to fiber shortening outweighs the effect of the compliant tendon.

In the extended Hill-type model where we added activation dependent stiffness to the muscle fibers modeling short-range-stiffness, the addition of a compliant tendon has a large influence on muscle-tendon stiffness as the muscle fibers become much stiffer than the tendon due to the added element (compare Figure 3 A vs 3B - stiffness). Note that even with the inclusion of a compliant tendon, modeling short-range stiffness results in about a five-fold increase in stiffness of the muscle-tendon unit at an activation level of 0.5 (Figure 3 A vs 3B, detail MT stiffness). We therefore expect simulations based on the muscle model with short-range-stiffness to change when including a compliant tendon. We speculate that for platform rotations in healthy individuals, postural sway might be lower as their strategy is to move the ankle in anti-phase to the platform which should be facilitated by a more compliant muscle-tendon unit. For vestibular loss subjects that try to follow the platform motion with their body a decrease in muscle-tendon stiffness might lead to lower levels of co-contraction, as it becomes less efficient, and higher postural sway. In the simulations of platform translations muscle co-contraction becomes a less efficient strategy at lower muscle tendon stiffness and would thus decrease for both healthy and vestibular loss subjects. This would then lead to an increase in postural sway for the translations.

*Figure 4 – simulated experiment for an isometric soleus muscle-tendon unit for different activation levels in the case of including a compliant tendon (CT) or a rigid tendon (RT) and in case of modelling the Hill-type muscle without (A) or with short-range-stiffness (B).*

6.      Methods. The problem formulation (eqs. 1-8) is very elegant and presented very clearly. Especially the incorporation of the state covariance into the path constraint is a great idea, with a meaningful parameter to specify a required probability of success.

Thank you.

7.      Supplementary material. Please delete any co-author comments that still appear in the margin.

Thank you, we have cleaned up the supplementary materials.

8.      Methods, equation (13). The equation of motion would be more clear if formulated in terms of absolute angle (relative to gravity) rather than ankle angle (relative to platform). As presented, the equation of motion is in a non-inertial reference frame, since qA is defined relative to the platform. With absolute angle, the w_SS,q and w_SS,qdd would not be needed here. The platform rotation would then have to accounted for in the relationship between absolute angle and muscle length, where the platform angle must be added to the absolute angle, to obtain ankle angle. I don’t think it would affect the nature of the problem (they are all noise terms) but it would be cleaner not to have to specify the angular acceleration noise.  But also see comment 15.

We have reformulated the equations of motion accordingly.

9.      Methods, equation (13).  The platform translation term in the equation of motion is incorrect, sin(qA) should be changed to cos(qA). As written, this term represents the effect of a vertical platform acceleration. Hopefully this is a typing error and the correct model was used in the computational work.

Thank you. This was a typo, indeed otherwise this term would disappear around the upright position. We realized it might not be obvious that all angles are expressed with respect to the vertical (or the axis perpendicular to the platform) such that sin(qA) is appropriate. we have clarified this point in lines 199 and 731.

10.   Line 585. When following my suggestion in comment 8, angular velocity of the platform would still need to be specified because it affects contractile element velocity. However, this would no longer be needed when series elasticity is used in the muscle model as suggested in comment 5. Only platform angle would be needed.

Although we have not added the series elasticity following the review, we think we would still need the velocity. The state derivative of the muscle-tendon unit position  depends on the muscle fiber velocity, and thus on the muscle tendon unit velocity.

11.   Line 585. If angle, angular velocity, and angular acceleration are all included in the model (which hopefully is not the case, see above), I think they can’t all be modeled independently as white noise. If the angular acceleration is white noise, the angular velocity would be the integral of white noise, i.e. Brownian motion which is not white noise.

You are correct that if we impose the translational acceleration to be Gaussian noise, this would be integrated, and the velocity and positions would not have Gaussian characteristics. In fact, this would be problematic as the uncertainty on the platform velocity and position would monotonically grow over time (as in a Brownian motion for the velocity). We therefore chose to impose all three (position, velocity and acceleration) of the platform as Gaussian. In practice, this might be impossible to achieve. But, one could imagine a platform controller that almost achieves such characteristics.

We added this intuition in the Methods section: “Note that we assumed here that the platform’s mean linear and angular position, velocity and acceleration were constant in time with constant variance. This differs from driving the platform acceleration with a zero-mean Gaussian input signal, which would lead to a monotonic increase of the velocity and position variance in time. Instead, we implicitly assumed that a more clever platform controller was used.”

12.   Methods. The noise terms are printed in red font, but you should probably not assume that this can be rendered like that in the published paper.

We will ask this to the journal editorial office. If this is not possible we will not indicate these terms.

13.   Methods, equation (9). The final term should have subscript TA rather than SOL.  But see also comment 1.

Thank you, we have made this correction.

14.   Methods, equation (10). It is odd that the short range stiffness is only dependent on the baseline activation.  If this is a mechanical muscle property, it can’t depend on how the activation is generated by neural circuits. It can only depend on the (total) activation. Hopefully this will be a moot point when comment 5 is addressed.

You are correct. The SRS should depend on a_tot = a_base + a. Since we also changed our control formulation to have a feedforward muscle excitation (baseline excitation) rather then formulating the baseline at the activation level, we achieve a more elegant formulation for the SRS force with the full activation: $F_{SRS}=k_{SRS}\cdot F_{iso,max}\cdot a\cdot\left( \frac{\tilde{l}_{m}-\tilde{l}_{m,mean}}{\tilde{l}_{m,opt}} \right)$

As a sidenote: having the full activation a, or only a_base will not change our results, and this was probably the reason why we didn’t formulate this correct in the first place.

- Note that the activation state has zero mean. This means that including it in the mean dynamics does not change the mean dynamics.
- The activation state ‘a’ has a variability (captured within the covariance matrix P) and to see whether including it in the SRS equation affects the covariance dynamics requires taking the derivative of the SRS equation wrt to the state.

15.   Methods, equation (14). If q_A is the ankle angle, the w_SS terms should not be in the proprioception model. However if my suggestion is followed and the equations of motion are formulated in absolute angle, the platform motion is correctly represented here.

We followed the suggestion and expressed the EOM in terms of the absolute angle

16.   Methods, equation (14). The feedback model has redundant parameters, and the optimization problem may not have a unique solution. For instance, e_SOL,fb = K11*(qA + w_p,q) + K13*(qA + w_SS,q + w_v,q) + ... = (K11+K13)*qA + ...   Therefore, K11 and K13 cannot be optimized indepedently, only their sum is relevant. This suggest that the problem must be simplified, which will also make the work more convincing. Similarly, some noise terms are mostly (sometimes exactly) additive, so only their sum is relevant. It may be more convincing to only have the noise terms that uniquely influence the solution. This will make the work appear less cluttered, and prevent the impression that results are dependent in numerous, somewhat arbitrary, assumptions about noise levels.

We disagree that K11 and K13 are redundant because they act on both the joint angle as well as the sensory noise terms, which are different for K11 and K13. The separation of K11 and K13 allows predicting sensory reweighting effects. The noise terms appear in the cost function in the expected effort terms and are differentially influenced by K11 and K13. In the example above, w_p,q does not equal w_SS,q + w_v,q so the optimization has a unique solution. Without sensory noise and only uncertainty on the states than indeed there is no need to separate K11 from K13.

17.   Line 614. Co-contraction index (CCI) was defined as the activation of the largest muscle. This does not resemble any other definitions of CCI that have been used in the literature. Always, the CCI is calculated from both activations: the agonist and the antagonist. And the CCI equation should always be symmetrical: if the two activations are switched, the CCI should remain the same. This definition only uses one muscle’s activation and should therefore not be named “co-contraction”.  Consider using a more standard definition of CCI.

We agree that this is not a standard definition, although it may boil down to the same thing for this simple problem. We now use the same CCI for both reaching and standing where we used the common formulation of Rudolph et al. 2000. in Knee Surgery, Sports Traumatology, Arthroscopy.

18.   The formatting of the Tables should be improved. All muscles should have a separate column. Sometimes columns are broken in two parts that are not vertically aligned.

done

19.   Table 2 has a note on the units of noise variance. Indeed the units are correct, but the explanation does not seem very helpful. Hopefully this can be improved. Would it be better to say: (m/s^2)^2 / Hz, and mention that this is represents the power spectral density? Readers may be more familiar with continuous-time noise described in the frequency domain.

This has been hard to present clearly indeed. Using the idea of PSD is a good suggestion and we now describe it in this way.

20.   Line 684 refers to Figure 2, but the model is shown in Figure 3. It is odd that results (Figure 2) are presented before the model.

fixed

21.   Musculoskeletal models for 2-DOF reaching will typically have six muscles, including two biarticular muscles. Such models are easily replicated from the literature or from Opensim.  Is there a particular reason for not doing this?

Based on your and other reviewers’ comments we repeated the simulations with an arm model driven by 6 muscles (Todorov 2003). This model differed substantially from our previous model in that the muscles had different moment arms and lengths. Also we now used the traditional (unadapted) muscle force-length characteristics as described in De Groote et al. (2016). In the previous model, these were adapted to make sure the muscles, that were a subset from the muscles in a more complex model, would operate on an acceptable region of the force-length curve.

In the main text we now only report the results with the new 6-muscle model.

The reach trajectories differ from the previous model (especially the perturbed trajectories for the circle and bar condition). In summary, the perturbed reach trajectories for the circle condition became more realistic as the overshoot that was not observed experimentally disappeared whereas the perturbed reach trajectories for the bar condition became less realistic as the overshoot that was observed experimentally also disappeared. For completeness, we added a comparison in the SI to show how the changes in the muscle model affect the predictions. We compared three models:

1. 6 muscle model (Todorov 2003,2005)
2. 4 muscle model (Todorov 2003,2005 but with biarticular muscles removed, other muscles unchanged)
3. 4 muscle model as described in the previous submission

Our reach trajectories with 6 muscles were not substantially different from simulations with 4 muscles (models 1 and 2).

Hence, differences in muscle parameters and not the addition of biarticular muscles explain the differences in reaching trajectories.

From these simulations, it is clear that muscle parameters play an important role.

We added the following to the discussion to refer to these additional simulations:

“…  We further explored the sensitivity of predicted reach trajectories to the presence of bi-articular muscles and muscle properties (see Supplement for details). Interestingly, simulated reach trajectories were robust against the removal of bi-articular muscles but sensitive to how mono-articular muscles were modeled. Using an alternative, physiologically plausible set of muscle properties yielded perturbed reach trajectories in the ‘bar’ condition that captured the experimentally observed overshoot. However, the overshoot was also predicted for perturbed reach trajectories in the ‘circle’ condition, where it was not observed experimentally. These exploratory results demonstrate both the potential of our framework to explore the effect of musculoskeletal properties on (perturbed) movement and the importance of accurate musculoskeletal models.”

This is the answer, the other part can be left out but is a good motivation to show that our results do not change when changing the formulation.
