# Supplementary material for: An approximate stochastic optimal control framework to simulate nonlinear neuro-musculoskeletal models in the presence of noise
Source: PLoS Comput Biol. 2022 Jun 8;18(6):e1009338. doi: 10.1371/journal.pcbi.1009338 (PMC9176817; doi:10.1371/journal.pcbi.1009338)
Supplement: Attachment — Submitted filename: Answers to reviewer.docx [file pcbi.1009338.s004.docx]

**Reviewer #1**
Please note that the paper still contains a number of typos that should be corrected before publication.

For instance, in the main text:
Fitt’s law -> Fitts’ law
‘to perform a point-to-point reaching movement over a distance of 25cm in 0.5s’ -> apparently it was rather 0.8s.
Corriolis -> Coriolis
Please check Eq. 25 (Fiso and activation seem to be inverted)
Eq. 2627 is misnumbered

And in the Supplementary Information:
missing transpose in Eq. 7 and below (for the term in C(t))
please correct the ‘dynamics wherethe controls:: ‘
‘reference trajectory ()’ is still mentioned

Thank you for pointing out these typos. We have corrected them.

**Reviewer #2:**

Minor comments:

1) Page 4. The references [35] [36] apparently should be [34] [35].

There was indeed something wrong with the reference numbers. We have set this straight throughout the whole document.

2) Page 4, three lines below the above references, suggest eliminating the first ‘the’ in the sentence so the sentence reads ‘This does not seem to yield an optimal solution as during the optimization . . .’

We followed this suggestion and adapted the text here.

3) The SRS acronym is still not clearly defined. The place to define it is on page 5 (near the middle of the page) were ‘short-range stiffness’ is first mentioned.

We now introduce the term short-range-stiffness and the acronym earlier as suggested by the reviewer.

4) Page 7. My previous review suggested that the Kandel reference (now reference [53]) should not be used for the explanations included on page 7 since this is a general textbook where it would be very hard to find what is discussed in this section. The original reference for the data shown in Kandel is the Peterka 2002 reference (now reference [36]). I also suggested that Figure 1A should include the proprioceptive weight measures shown for vestibular loss (VL) subjects. These VL proprioceptive weights are shown in Figure 10 of [36]. The authors apparently did not understand that Figure 10 does show proprioceptive weight measures from four VL subjects obtained in an eyes closed, surface-tilt stimulus condition that is relevant to the discussion in this section (as well as other conditions that are not relevant here). Another reference that more clearly presents that Peterka 2002 weights for the eyes closed, surface tilt condition is in Figure 2.5A of the reference:

Peterka, R.J., Sensory integration for human balance control. Handbook of Clinical Neurology, Vol 159 (3rd series) Balance, Gait and Falls, B.L. Day and S.R. Lord editors, 159:27-42, 2018.

Thank you for pointing us to this reference. This is indeed much easier for the reader to find the information, and helped us to better understand and implement the previously made comment.

We added these datapoints to the relevant figure to show how in experiments with vestibular loss subjects when standing upright in an eyes-closed condition the proprioceptive feedback gains are close to 1. We changed the caption of figure 1 to describe the changes in the figure, and we added explanation in the results:

*“In the case of vestibular loss, the relative weighting of proprioceptive information was 100% in simulation (Figure 1– A – simulations, vestibular loss) and nearly 100% in experiments (Figure 1– A – experiments, vestibular loss) throughout platform rotations. Peterka et al. determined sensory weighting was found by identification of the feedback gains* $\tilde{K}_{proprio},\tilde{K}_{vest},\tilde{K}_{vis}$ *of a linear model based on collected sway data, with* $\tilde{K}_{vest}=0$ *for vestibular loss subjects [52]. In this study [52], the identified* $\tilde{K}_{vis}$ *in the eyes-closed condition were close to but not exactly 0, explaining the small deviations from 100% proprioceptive weighting in this condition.*”

5) Page 9 near the middle of the page, the sentence starting ‘Similar to the experiments, . . ‘. Suggest to use ‘ . . . for the obstacle task compared to the circle task as the second part . . .’

We followed this suggestion and adapted the text here.

6) Page 17, equation (11). This equation included the variable ‘a’ but the next line refers to ‘a_base’.

We changed ‘a_base’ to ‘a’

7) Page 23 just before the [81] reference. Units should be (Nm)^2/Hz.

Thank you, we fixed this typo.

8) Page 5 of ‘Supplementary Information’ document. At the bottom of page 5 there are 5 equations shown but the equation numbers include equation (4) for which there is no actual equation shown. Assuming equation (4) should not exist, its elimination would affect subsequent equation labeling.

Thank you, we fixed this typo.

9) Page 6 of ‘Supplementary Information’ document. Should the gamma variable in equation (10) include a ‘i’ subscript as in equation (8) in the main manuscript?

Yes it should, we added this subscript to be consistent with the main manuscript.

10) Page 8 of ‘Supplementary Information’ document. Between equations (27) and (28) there is the ‘s.t.’ abbreviation. Better to spell out ‘subject to’.

We followed this suggestion and adapted the text here.

11) Page 10 of ‘Supplementary Information’ document. Under point (2) of the ‘We observed the following problems.’ section, better to say ‘. . . must be chosen to be smaller, but . . .’

We followed this suggestion and adapted the text here.
